# Addressing the severity and intensity of poverty in Sub-Saharan Africa: how relevant is the ICT and financial development pathway?

**DOI:** 10.1016/j.heliyon.2021.e08156

**Published:** 2021-10-12

**Authors:** Isaac Kwesi Ofori, Mark Kojo Armah, Francis Taale, Pamela Efua Ofori

**Affiliations:** a Department of Data Science and Economic Policy, School of Economics, University of Cape Coast, Cape Coast, Ghana; b Department of Economic Studies, School of Economics, University of Cape Coast, Cape Coast, Ghana; c Department of Economics, University of Insubria, Via Monte Generoso, 71, 21100, Varese, Italy

**Keywords:** Africa, Financial access, Financial development, ICTs, ICT access, Inequality, Poverty

## Abstract

The study examines the effectiveness of ICT diffusion and financial development in reducing the severity and intensity of poverty in Sub-Saharan Africa (SSA). Using data from the World Development Indicators and the Global Consumption and Income Project (1980–2019), we provide evidence, robust to several specifications from the dynamic system GMM and the panel corrected standard errors estimation techniques, to show that, compared to financial access, ICT usage, and ICT access, ICT skills is remarkable in reducing both the severity and intensity of poverty. The results further revealed that, though ICT skills reduce poverty, the effect is more pronounced in the presence of enhanced financial development. Policy recommendations are provided in line with the region's green growth agenda and technological progress.

## Teaser

Financial Access, Financial Development, ICT, Inequality, Poverty, Africa

## 1 Introduction

Before the coronavirus disease (COVID-19) struck in late 2019, growth in Sub-Saharan Africa (SSA) compared favourably to that of the world. In fact, growth in the region averaged 3.2 per cent in 2018 and 3.4 per cent in 2019 compared to the world's average of 3.5 per cent and 2.8 per cent in respective periods (IMF, 2020a). Despite its multifaceted dismal effects, the coronavirus pandemic has laid bare the porous growth trajectories of the region in recent times. Further, notwithstanding the deepening of efforts by African leaders to foster shared prosperity as enshrined in *The Africa We Want*^1^ by 2063, academic and political discourses in the SSA have largely centred on economic growth (Ofori, 2021; Ofori and Asongu, 2021a; Greenwald and Stiglitz, 2013). However, in the wake of the coronavirus pandemic, attention has turned considerably towards building shared growth, with the agenda of *Leaving No One Behind* taking centre stage. Indeed, the plummeting of the region into a record 1.9 per cent contraction in economic activity in 2020 (IMF, 2020a; World Bank, 2020a) can be traced to the fact that the region is highly informal, unequal and disadvantaged (World Bank, 2020b; Ravallion and Chen, 2019).

Particularly, information gleaned from World Bank (2020b), ILO (2020a), and OECD (2020a) shows that the pandemic has eroded hard-fought gains chalked over the past few years on Sustainable Development Goals^2^ 1, 8 and 10. More crippling is the bleak socioeconomic outlook of the region, specifically, the projection of an upsurge in both extreme poverty and income inequality levels. On poverty, the World Bank (2020b) estimates that the pandemic pushed a staggering 88 – 115 million people back into the extreme poverty bracket in 2020, with at least half of this number residing in SSA alone. On top of this is the projection of a further swell in this number by 23 – 35 million in 2021. It is also projected that an astonishing 87 per cent of the world's poorest people will reside in SSA by 2030 if current economic challenges are not tackled head-on^3^. Moreover, income inequality could rise due to the slow recovery of informal activities, job losses, food price shocks, and low social protection in the developing world (Kovacevic and Jahic, 2020; World Bank, 2020b; ILO Newsroom ILO, 2020b).

The implications of welfare reversals in unequal and disadvantaged societies can be found in Pickett and Wilkinson (2015, 2010) who argue that such developments have deleterious effects on the quality of life, education, social protection efforts, and mortality. The challenge facing policymakers interested in the SSA growth agenda is thus enormous. Even before the COVID-19 pandemic was the region's hydra-headed problems of climate change^4^ and food security, unemployment, and geopolitical frailties^5^. Going forward, building a sustainable and all-inclusive SSA should take into consideration the *green growth* strategies^6^ and technological progress. This is where policy recommendations are needed but comprehensive empirical contributions are hard to find.

In this study, we identify two channels that are in line with SSA's *green growth* strategies– information and communication technologies (ICTs) diffusion, and financial development that can be targeted due to their human and socioeconomic development strengths (Ofori and Asongu, 2021b; Andrés et al., 2017). If the power of financial institutions and ICTs in driving and sustaining economic activity had ever been in doubt, the pandemic refuted it all. In the heat of the pandemic, decisionmakers relied on financial institutions for social protection– reaching out to vulnerable households, incentivising frontline workers, and boosting online transactions. A blessing from the pandemic is that it amplified the usefulness of ICTs. For instance, during lockdowns and/or ban on social gatherings, ICTs facilitated the settlements of bills, e-banking, ordering of consumables, access to educational services through E-learning, preservation of jobs as well as access to health information and entertainment. Also, quite recently, in the context of SSA, opportunities relating to employment and payment for consumer goods and services are becoming available online via financial institutions or mobile money service providers.

Despite these developments, the lacuna in the literature is that contributions exploring the possible synergistic relationships between ICTs and financial development in bidding down SSA's persistent problems of high intensity and severity of poverty are hard to find. Indeed, empirical works in line with our argument only estimate the direct and/or indirect pathway effects of financial development, financial access, and ICTs on economic growth or poverty intensity, clearly losing tabs on the severity of poverty (Bolarinwa et al., 2021; Cheng et al., 2021; Opoku et al., 2019; Peprah et al., 2019; Latif et al., 2018; Das et al., 2018; Sassi and Goaied, 2013; Shamim, 2007). The purpose of this paper is thus twofold. First, we explore the effects of ICT diffusion and financial development on the intensity and severity of poverty in SSA. Second, we explore the joint effects of ICTs and financial development on the intensity and severity^7^ of poverty in SSA. The attendant hypotheses are thus:

1. $H_{1}$: ICTs have suppressing effects on the severity and intensity of poverty in SSA.
2. $H_{1}$: Financial development and financial access amplify the suppressing effects of ICTs on the severity and intensity of poverty in SSA.

The rest of the paper is organised as follows: the next section is dedicated to the theoretical link between poverty, ICTs and financial development. Section 3 presents the methodological foundation of the paper. The results and discussions are presented in Section 4 while Section 5 concludes with some policy recommendations.

## 2 The theoretical link between ICT, financial development and poverty

The theoretical foundation of this paper draws on two streams of ideas– the neoclassical models of economic development and the Sustainable Livelihoods Approach (SLA). The former illustrates a link between ICTs and the participation of vulnerable groups in decent economic activity (Kwan and Chiu, 2015). The neoclassical theory posits that ICTs are instrumental in aiding poor countries' transition out of endemic poverty, evidence of which is the case of China, Hong Kong, and Japan. The SLA also denotes the different linkages between livelihood assets, institutions and policies as well as people's livelihood outcomes (Messer and Townsley, 2003). The SLA framework rests on Sen (1999) notion^8^ of the set of *functionings* and *doings* in people's capabilities. The approach fundamentally indicates how economic agents can create opportunities for themselves by drawing on assets or productive materials at their disposal. As Gigler (2011) reckons, ICTs are a complete array of contemporary assets^9^ with or through which people can create opportunities for themselves by participating in various socioeconomic activities. It is in the context of this and the flexibility of the SLA concept in analysing the vulnerability, intensity, and severity of poverty that ICT is incorporated into the framework (Duncombe, 2006).

The link between financial development and the creation of opportunities for the masses also stems from the scholarly works of Beck (2012), Demirgüç-Kunt and Levine (2009), McKinnon (1973), Shaw (1973), and King and Levine (1993). The authors highlight the significance of a burgeoning, efficient, dynamic and innovative financial sector in resource allocation and the eventual development of an economy. There is also the evidence that, compared to other sectors such as manufacturing and hospitality, the financial sector tops in terms of the depth and application of ICTs (Shamim, 2007; Allen et al., 2001). In the developing world, where administrative and structural inefficiencies impede financial development and its growth-lubricating effects, ICT diffusion can be used to achieve operational efficiency. Indeed, ICT diffusion can facilitate financial competition and inclusion while enhancing long-run growth prospects^10^ by reducing both the processing and information costs of financial players (Asongu and Odhiambo, 2020; Asongu and Nwachukwu, 2018; Asongu et al., 2019; Muto and Yamano, 2009; Shamim, 2007).

### 2.1 Literature survey on ICTs, financial development and poverty

Zahonogo (2017) applied the system GMM estimation technique on a panel of 42 SSA countries for the period 1980–2012 to show that financial development drives poverty reduction. Particularly, the results indicate that there is a 1.19% threshold level required for financial development to have a dampening effect on poverty. Using an unbalanced panel of 60 developing countries for the period 1985–2008, Rashid and Intartaglia (2017) also used the two-step system GMM to report that financial development is robust in reducing absolute poverty. On the contrary, Seven and Coskun (2016) explored whether financial development channels (the bank and stock market) are effective for reducing income inequality and poverty in 45 emerging economies. The study, which covered the period 1987–2011 finds that both financial development channels do not matter for addressing inequality and poverty. Boukhatem (2016) employed the GMM technique on a panel of 67 low- and middle-income countries from 1986 – 2012 and finds that financial development is a key channel for alleviating poverty. Boukhatem further reports that financial development is less relevant in bidding down poverty in the presence of financial instability.

Similarly, Ngongang (2015) examined the empirical link between economic growth and financial development in 21 SSA countries and found that financial development directly induces economic growth and by extension, poverty. Batrancea et al. (2021) also used 7 countries over the period 1990–2019 to explore the determinants of economic growth, which is essential for income growth and distribution. The authors provide evidence from the fixed effect and random effect estimators to conclude that economic growth is mainly influenced by bank capital to assets ratio. Likewise, Batrancea et al. (2020) employed panel data of 3 countries from 1970-2018 to conclude that, the financial sector plays a key role in the areas of green investment, economic growth and poverty alleviation. Yilmaz and Koyuncu (2018) also analysed an unbalanced panel data for 182 countries for the period 2000 – 2013 and found evidence from the fixed effect and random effect estimators that ICTs matter for reducing poverty and inequality. Particularly, the study shows that, among all ICT diffusion indicators, the broadening of internet access plays a key role in poverty and income inequality alleviation. Similar finding is reported by Alimi and Okunade (2020) who maintained that ICT diffusion is an important driver of poverty reduction in SSA.

A study conducted by Mushtaq and Bruneau (2019) also focussed on the impact of ICT in poverty alleviation. The study relied on a panel dataset of 61 countries from 2001 to 2012 and the Quintile and instrumental variable regressions to conclude that financial enhancement is a pathway through which ICT diffusion alleviates poverty and inequality. Rewilak (2017) also examines the poverty effects of financial access and deepening in middle income and poorest countries over the period 2004–2015. The author applies the fixed effect estimator and finds that compared to financial access, financial deepening is greatest in reducing poverty. Similarly, Boukhatem (2016) draws on data for a panel of 67 low- and middle-income countries for the period 1986 – 2012 and finds evidence from the system GMM to show that financial development is a key contributor to poverty reduction. Further, De Haan et al. (2021) explore the effects of financial development on poverty for 84 countries over the period 1975 – 2014. The authors provide strong evidence from the fixed effect estimator to show that while financial development does not have a significant effect on poverty intensity, economic growth proved effective.

### 2.2 Overview of ICTs, financial development, and poverty in Sub-Saharan Africa

**Figure 1:**
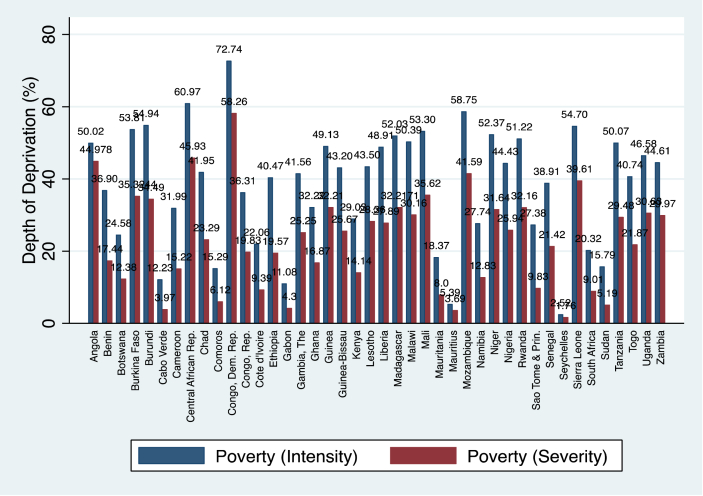
Average poverty intensity and severity in SSA, 1984–2019.

If there is any region of the world in need of attention in terms of policy recommendations in addressing poverty and inequality, then it is the SSA. To begin with, the region is already grappling with several challenges believed to be caused by climate change and geographical fragilities. Secondly, aside the erosion of the welfare gains imposed implicitly by the COVID-19, the region is also projected to experience a rise in vulnerable employment and unemployment (ILO Newsroom ILO, 2020b). Though several countries, markedly, Ghana, Angola, Rwanda, Botswana, Lesotho, and Ethiopia boast of achieving high growth rates and halving extreme poverty levels in the past three decades, poverty levels in most SSA countries are still high. To put the study into perspective, Figure 1 is presented to show the level of within-country poverty intensity and severity in SSA over the study period.

As is evident from Figure 1, poverty intensity and severity levels are high in countries like Burundi, Congo DR., Central African Republic, Niger, Mozambique and Sierra Leone.

**Figure 2:**
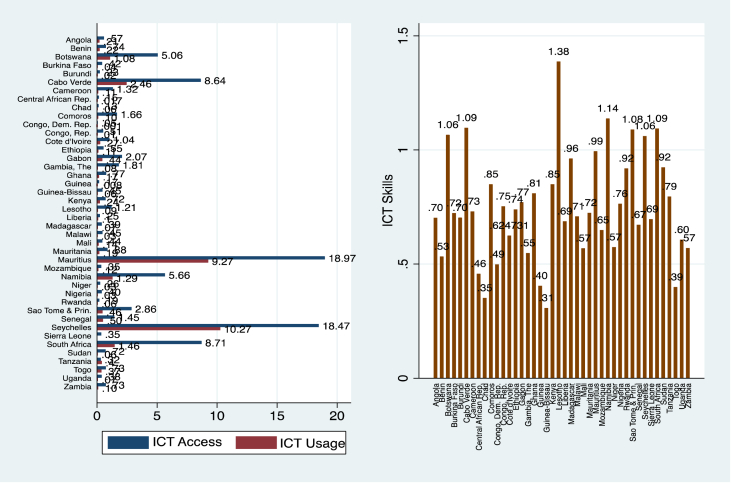
Average ICT access, usage (left), and ICT skills (right) in SSA, 1984–2019.

**Figure A.1:**
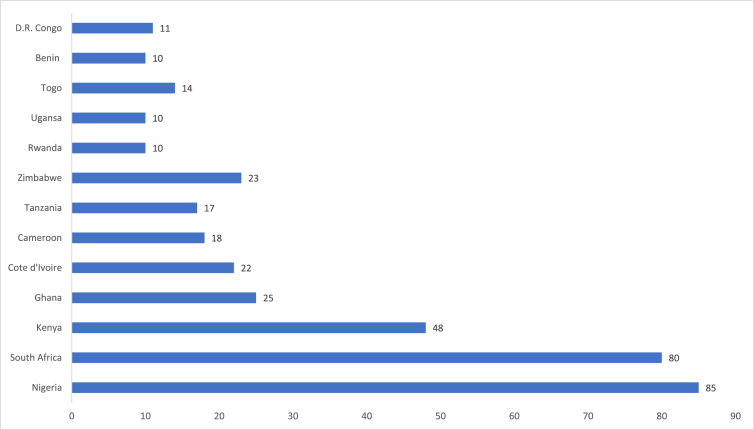
Major Tech-Hub Countries in Sub-Saharan Africa. Source: GSM Association Data, 2021.

The world is ever-changing, driven largely by ICTs. Indeed, as Castells (1999) puts it, the current era is *information age* where lack of ICT in itself is social exclusion/deprivation, likening it to lack of access to electricity in the *industrial age*. However, sceptics question the role of ICTs in poverty eradication citing cost/affordability, adaptability challenges, poor infrastructure in the developing world, and possible inequality- and unemployment-inducing effects (Chowdhury, 2000). These arguments have, to some extent, been rebutted by others who argue that, in countries where social transfers are low, unemployment is high, and resources are constrained, ICTs offer a good medium to leapfrog development, tackle poverty, and enhance inclusiveness^11^ (Asongu, 2017; Asongu and Le Roux, 2017; Grace et al., 2003; Kenny, 2002; Brown, 2001; Wolf, 2001). In fact, the SSA is home to the world's youthful and innovative population. There is also the abundance of natural resources and unmet gaps for infrastructure, and a major recipient of foreign direct investment (FDI) from Europe and Asia (UNCTAD, 2019). Two key developments are glimmers of hope in addressing the region's growing poverty through ICT diffusion/innovation. First is the rise in ICT access, ICT skills, and ICT usage, which as we show in Figure 2 is expanding rapidly in SSA. Second is the springing up of technology/innovation-hubs^12^ (tech-hubs) in countries such as South Africa, Nigeria, Kenya, Ghana, and Cote d’Ivoire (Figure A.1), connecting young programmers, designers, entrepreneurs, and investors for the cultivation and nurturing of ideas.

For instance, the Global System for Mobile Communication Association reports a momentous rise in tech-hubs development in SSA– from 314 in 2016 to 442 in 2017 and 643 in 2019. At the backbone of resilient tech-hubs, which can turn the young and creative minds into economic development process is financial access. We reckon that if prioritised with financial access and development, the current ICT wave in SSA can offer limitless shared opportunities by (1) creating green wealth through access to greater markets like one offered by the African Continental Free Trade Area (AfCFTA), (2) enhancing access to education and information, (3) encouraging innovation transfer, relationship and network formation, and (4) fostering social inclusion.

**Figure A.2:**
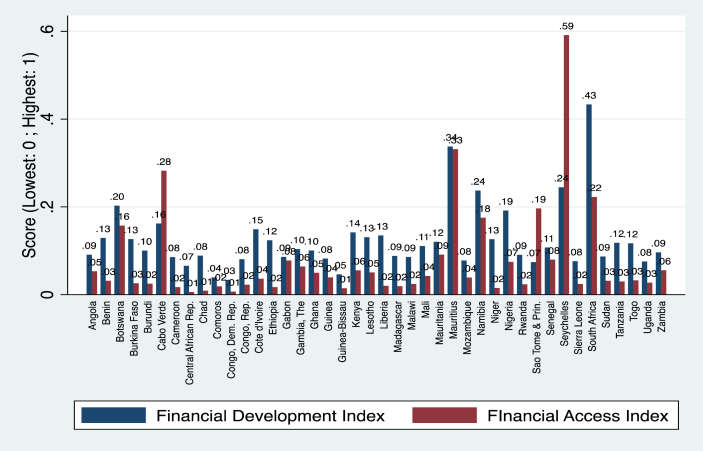
Average Financial Development and Financial Access in SSA, 1984 – 2019.

**Figure A.3:**
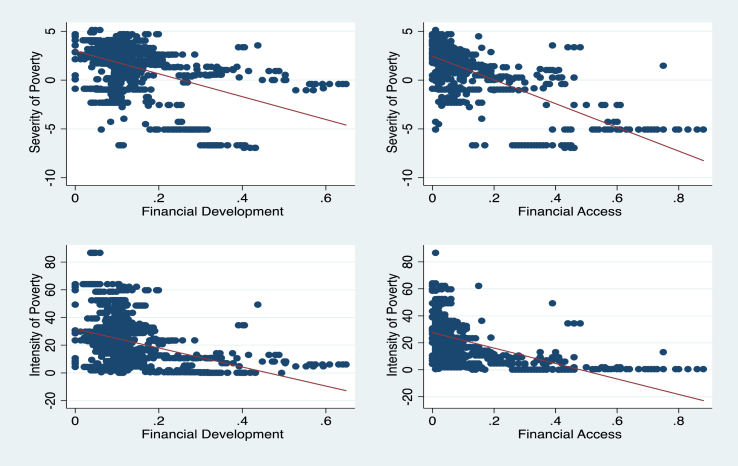
Financial Development/Access – Poverty Severity/Intensity Nexus.

Despite lags in some countries such as Sudan, Chad, the Central African Republic, and Niger, financial access and development are also growing steadily in SSA (see Figure A.2). In settings like this, complementarities between ICT diffusion and financial development can be a gamechanger in addressing the severity and intensity of poverty. The graphical relationships between our poverty indicators (severity and intensity) and financial development we show in Figure A.3 are in line with our empirical findings, which as we show in Section 4 provide evidence for our objectives.

## 3 Data and methodology

### 3.1 Data

The dataset underpinning this study spans 1980 – 2019 on 42 SSA countries^13^. Our focus on the intensity and severity of poverty stems from massive welfare setbacks triggered by COVID-19 and the renewed calls for African leaders to foster shared prosperity (Africa Union, 2015). We use the international poverty gap (US$1.90) as our indicator for poverty intensity^14^. We draw data on poverty intensity from the World Bank's Poverty and Equity Database (World Bank, 2021a). The squared poverty gap index, which is calculated following Foster et al. (1984) is used to measure the severity of poverty. We evaluate the robustness of our results on poverty severity using the Palma ratio, which is also sourced from the Global Consumption and Income Project (Lahoti et al., 2016). Likewise, the robustness on poverty intensity was checked using the middle-income poverty gap of US$3.20. We point out that missing observations in the poverty intensity measures were addressed with information from the Global Consumption and Income Project (Lahoti et al., 2016) and Our World in Data (Roser and Ortiz-Ospina, 2013).

Measures of financial development and financial access were taken from the International Monetary Fund's Financial Development Index Database (Svirydzenka, 2016). Following the International Telecommunication Union, we focus on three indicators of ICTs– *access, usage,* and *skills*. Our interest in ICTs follows contemporary arguments that ICTs are valuable assets^15^ with or through which economic agents can create opportunities for themselves or access opportunities (Adams and Akobeng, 2021). Data on all ICT indicators are also sourced from the WDI (World Bank, 2021b).

For controls, we consider variables such as foreign aid, economic growth, vulnerable employment, economic globalisation, and social inclusion. Foreign aid is proxied by the net official development assistance as per cent of gross domestic product (GDP). This variable is used to capture the contribution of international bodies and foreign governments in poverty eradication (OECD, 2018; Boateng and Adom, 2019; UNDP, 2017). Vulnerable employment allows us to capture the structure of the real sector in the selected countries (Ofori and Asongu, 2021a). While economic growth is used to denote the contribution of economic expansion in poverty alleviation through the creation of fiscal space for enhanced social protection and the creation of opportunities (Lustig et al., 2019), we use economic globalisation and social inclusion to capture the contribution of trade, FDI, capital flows, and institutions in the eradication of poverty and its severity. Data on economic globalisation is sourced from the Konjunkturforschungsstelle (KOF) globalisation^16^ index (Dreher, 2006; Gygli et al., 2019) with all other controls coming from the WDI (World Bank, 2021b). The description of the variables is provided in Table 1.

**Table 1:** Variable description and sources.

Variables	Description	Data Source
***Outcome variables***		
Poverty severity	Squared poverty gap index	Generated
Palma ratio	The ratio of the share of the top 10% to that of the bottom 40 % in the population	GCIP
Poverty intensity	Poverty gap at $1.90 a day (2011 PPP)	PED, OWID
Poverty gap	Poverty gap at $3.20 a day (2011 PPP)	PED, OWID
***Variables of interest***		
Financial development	Financial development index capturing the efficiency, access, and depth of the financial institutions and markets	Findex
Financial access	Financial institutions access capturing the access of people to financial institutions	Findex
ICT access	Fixed telephone subscriptions (per 100 people)	WDI
ICT use	Fixed broadband subscriptions (per 100 people)	WDI
ICT skills	Gross secondary school enrolment gender parity (ratio)	WDI
***Control variables***		
Social inclusion	Country policy and institutional assessment score indicating the effectiveness of social inclusion institutions	WDI
Economic globalisation	Captures trade in goods and services; customs duties, taxes and trade restrictions; capital account openness and international investment agreements.	KOF
Economic growth	Annual growth in real GDP	WDI
Foreign aid	Net official development assistance (%GDP)	WDI
Vulnerable employment	Total contributing family and own-account workers as a share of total employment	WDI

Note: WDI is World Development Indicators; Findex is IMF's Financial Development Index; KOF is Konjunkturforschungsstelle index, and PED is Poverty and Equity Database; OWID is Our World In Data.

### 3.2 Estimation strategy

The theoretical strength of this paper rests on the neoclassical models of economic development (Kwan and Chiu, 2015), the SLA (Messer and Townsley, 2003) and the established link between ICTs and financial development toward the creation of opportunities (Asongu and Nwachukwu, 2018; Asongu, 2013; Muto and Yamano, 2009; Shamim, 2007). The empirical rigour of this paper begins with the specification of baseline models where for both outcome variables (poverty intensity and poverty severity), neither ICT indicators nor financial development (and financial access) enters the models. Per our hypothesized pathways, we proceed with the stepwise introduction of financial development, ICTs as well as their interaction terms in the models. We also interact the components of ICTs and financial access. This is strictly from policy sense because it is financial access that denotes the masses’ direct access to resources from financial institutions (IMF & World Bank, 2020). We specify our baseline model for poverty severity as follows:

(1) $$ln\left(povsev_{it}\right)=\alpha_{0}+\gamma_{1}ln\left(povsev_{it-1}\right)+\gamma_{2}ln\left(ecog_{it}\right)\;+\gamma_{3}ln\left(growth_{it}\right)+\gamma_{4}ln\left(faid_{it}\right)\;+\gamma_{5}ln\left(vul_{it}\right)\;+\gamma_{6}ln\left(socinc_{it}\right)\;+\;\varepsilon_{it}$$

We incorporate the interaction terms for ICTs and financial development into Eq. (1) to obtain Eq. (2):

(2) $$ln\left(povsev_{it}\right)=\alpha_{0}+\gamma_{1}ln\left(povsev_{it-1}\right)+\gamma_{2}ln\left(ecog_{it}\right)\;+\gamma_{3}ln\left(growth_{it}\right)+\gamma_{4}ln\left(faid_{it}\right)\;+\gamma_{5}ln\left(vul_{it}\right)\;+\gamma_{6}ln\left(socinc_{it}\right)\;+\gamma_{7}ln\left(icts_{it}\right)+\gamma_{8}ln\left(fdev_{it}\right)+\gamma_{9}ln\left(icts_{it}\times fdev_{it}\;\right)\;+\;\varepsilon_{it}$$

Likewise, we specify the baseline model for our poverty intensity model as:

(3) $$ln\left(povint_{it}\right)=\alpha_{0}+\omega_{1}ln\left(povint_{it-1}\right)+\omega_{2}ln\left(ecog_{it}\right)\;+\omega_{3}ln\left(growth_{it}\right)+\omega_{4}ln\left(faid_{it}\right)\;+\omega_{5}ln\left(vul_{it}\right)\;+\omega_{6}ln\left(socinc_{it}\right)\;+\;\varepsilon_{it}$$

The attendant main poverty intensity model when our ICT dynamics, financial development and access are included is thus a modification of Eq. (3) to obtain Eq. (4)

(4) $$ln\left(povint_{it}\right)=\alpha_{0}+\omega_{1}ln\left(povint_{it-1}\right)+\omega_{2}ln\left(ecog_{it}\right)\;+\omega_{3}ln\left(growth_{it}\right)+\omega_{4}ln\left(faid_{it}\right)\;+\omega_{5}ln\left(vul_{it}\right)\;+\omega_{6}ln\left(socinc_{it}\right)\;+\omega_{7}ln\left(icts_{it}\right)+\omega_{8}ln\left(fdev_{it}\right)+\omega_{9}ln\left(icts_{it}\times fdev_{it}\;\right)\;+\;\varepsilon_{it}$$

where from Eqs. 1 – 4, povsev is poverty severity; povint is poverty intensity; ecog is economic globalisation; growth is economic growth; faid is foreign aid; vul is vulnerable employment; socinc is social inclusion score; and icts is our ICT diffusion indicator for ICT access, ICT usage and ICT skills. Also, fdev is financial development index; icts×fdev is the interaction term for financial development and ICT indicators; *ln* is the natural logarithm.

It is imperative to note that in models 1 – 4, $\varepsilon_{it}=\varepsilon_{i}+\vartheta_{t}+\mu_{it}$; $\varepsilon_{i}$ is unobserved country-specific fixed effects; $\vartheta_{t}$ is the time effects, and $\mu_{it}$ is the idiosyncratic error term. There is a suspicion of endogeneity in models (1) to (4) due to the introduction of the lags of the outcome variables (i.e., povsev or povint) in the respective models. In the poverty model, for instance, the endogeneity arises as $povsev_{it-1}$ depends on $\varepsilon_{it-1}$, which is a function of the country-specific effect $\varepsilon_{i}$. To the extent that unresolved endogeneity concerns can render our inferences flawed, we address it using the dynamic system GMM technique^17^ (Arellano and Bover, 1995). The attendant net effects from the interaction terms for ICTs and financial development on both the severity and intensity of poverty from Eqs. (2) and (4) are expressed respectively as:

(5) $$\frac{\partial \left(ln\left(povsev\right)\right)}{\partial \left(icts\right)}=\gamma_{7}+\gamma_{9}\bar{fdev}$$

(6) $$\frac{\partial (ln\left(povint\right))}{\partial \left(icts\right)}=\omega_{7}+\omega_{9}\bar{fdev}$$

where $\bar{fdev}$ is the average financial development index. For brevity, we indicate that the financial access-ICT joint effects and the attendant net effects are computed^18^ following specifications in Eqs. (2), (4), (5), and (6). Finally, we apply the panel corrected standard errors estimation (PCSE) technique as well to evaluate the robustness/persistence of our hypothesized relationships. We opt for the PCSE since it provides robust estimates in the presence of possible correlation across our panels (Beck and Katz, 2011).

### 3.3 Construction of poverty severity (PS) index

Our outcome variable, poverty severity (squared poverty gap index) is calculated following the Foster-Greer-Thorbecke approach. In doing so, we average the poverty gaps relative to the poverty line/headcount (US$1.90), where the weights used are the within-country poverty gaps of US$1.90. The poverty severity index is expressed in Eq. (7) as:

(7) $$PS_{\alpha }=\frac{1}{N}\overset{N}{\underset{i=1}{\sum }}{\left(\frac{G_{i}}{z}\right)}^{\alpha },\;\alpha \geq 0$$

where *α* denotes the sensitivity of $PS_{\alpha }$ to poverty, *z* is the poverty headcount (US$1.90), and $G_{i}$ is the within-country poverty gap. It follows that if α = 0, $PS_{0}$ converges to the poverty head-count measure. Likewise, if α = 1, the index becomes the poverty gap index ($PS_{1})$, while $PS_{2}$ becomes the poverty severity index if α = 2. This is interpreted to mean that for α > 0, $PS_{2}$ is strictly decreasing in the living standard of the poor.

## 4 Results and discussion

### 4.1 Summary statistics

We provide the overview of the dataset by presenting the summary statistics in Table 2.

**Table 2:** Summary statistics.

Variable	N	Mean	Std. Dev.	Minimum	Maximum	Kurtosis	Skewness
***Dependent variables***							
Poverty severity	1,680	16.575	22.441	0.000	169.2993	11.876	2.632
Poverty intensity	1,680	23.180	16.906	0.000	86.700	3.205	0.781
Palma ratio	1,680	7.283	3.750	0.000	30.065	17.481	3.426
Poverty gap (US$3.20)	1,680	38.299	19.265	.4.000	86.700	2.357	-0.102
***Variables of interest***							
Financial development	1,680	0.124	0.089	0.000	0.6480	10.179	2.228
Financial access	1,680	0.076	0.128	0.000	0.880	13.991	3.160
ICT access	1,492	2.178	4.855	0.000	34.273	19.981	3.962
ICT use	1,492	0.836	2.852	0.000	27.603	37.924	5.617
ICT skills	1,680	0.772	0.274	0.180	1.527	2.457	0.167
***Control variables***							
Economic globalisation	1,680	40.048	11.263	0.000	85.299	3.865	0.359
Social inclusion	1,492	3.162	0.474	0.000	4.300	3.653	-0.279
Vulnerable employment	1,680	70.927	22.867	8.826	94.759	3.409	-1.207
Foreign aid	1,680	11.362	11.556	-0.251	94.946	11.391	2.445
GDP growth	1,680	3.590	5.210	-50.248	35.224	16.32-	-1.313

The data shows an average poverty intensity and severity of 23.13 and 16.57 respectively over the study period. Though the average severity of poverty is less than the intensity, it is very high requiring policy attention. Likewise, the mean for financial development is 0.12. The data also unveils a moderately high foreign aid of 11.36 per cent. ICT access and ICT usage also averaged 2.17 and 0.83, respectively. The pairwise correlation between the variables is presented in Table A.1.

### 4.2 Bivariate results on the effects of financial development and ICTs on the severity and intensity of poverty in SSA

In this section, we focus on the presentation and discussion of the results. We start with the presentation of our results with a test on the stationarity of the variables. Results from both the cross-sectionally augmented Dickey-Fuller, and the Cross-sectionally Augmented Im, Pesaran, Shin unit root tests in Table A.2 indicate that all the variables are stationary, providing impetus for sound regression analysis. We proceed to investigate the bivariate relationship between our ICT indicators, and financial development on both the severity and intensity of poverty in SSA. The results as presented in Table A.3 show that both financial development and financial access are remarkable in reducing the intensity and severity of poverty in SSA. On ICTs, though all the components are negative and statistically significant, we find that ICT skills is more effective in reducing both the intensity and severity of poverty.

### 4.3 System GMM results on the effects of financial development and ICTs on the severity of poverty in SSA

Our results on poverty severity are based on Eq. (1) for the baseline estimates and Eq. (2) for that of the main results. The baseline results in Column 1 show that economic growth, social inclusion and economic globalisation are significant drivers of the severity of poverty in SSA. Albeit not statistically significant, both vulnerable employment and foreign aid carry the *a priori* signs.

For the first objective, we find that both financial development and financial access have a negative relationship with the severity of poverty in SSA (see Columns 2 and 3 respectively). On the unconditional effects of our ICT dynamics, we provide strong empirical evidence to show that all the ICTs matter for reducing the severity of poverty in SSA. In specifics, we find that for every 1 per cent improvement in ICT access and skills, the severity of poverty reduces by 0.005 and 0.13, respectively (Columns 4 and 6). Further, we provide strong empirical evidence to show that ICT usage has a 0.01 suppressing effect on poverty severity in SSA. These results provide evidence for the propositions that expanding ICT skills can enhance the capability of people to create opportunities for themselves and offer concrete means of transitioning out of poverty. Indeed, our results provide optimism about the future of education and skills in shaping opportunities, reducing inequalities and poverty. With growing tech-hubs in countries like Nigeria, Kenya, Ghana, and South Africa as well as favourable ecosystems to start-ups in the form of large markets, good network and internet coverage, ICT skills, access and usage can spur shared prosperity through ideation and product development. Additionally, the rise in tech-hubs means that ICT diffusion can aid SSA's youthful population realise their innovative or entrepreneurial ideas and contribute meaningfully to national development. The economic impacts created through ICT diffusion offer policymakers concrete opportunities for addressing welfare issues such as poverty severity.

We find empirical support for our second objective as well. All our interaction terms are negative, signifying that complementary policies on financial development in general, financial access and ICTs matter for reducing the severity of poverty in SSA. The uniqueness of our results is that, of all our ICT dynamics, it is ICT skills that matter most for forming relevant synergies with financial development and financial access on reducing poverty severity. First, the net effect of enhancing ICT skills given the current average financial development in SSA is –0.47. This is computed from Eq. (5) as:

$$\frac{\partial \left(povsev\right)}{\partial \left(icts\right)}=-0.2153+\left(-2.0551\times \bar{fdev}\right)$$

where -0.2153 is the unconditional effect of ICT skills; -2.0551 is the conditional effect of ICT skills; and $\bar{fdev}$ denotes a constant term for the average financial development, which is 0.124 as apparent in Table 2.

$$\frac{\partial \left(povsev\right)}{\partial \left(ICT\left(skills\right)\right)}=-0.2153+\left(-2.055\times 0.124\right)=-0.470$$

Similarly, we compute the financial access and ICT skills net effect in Columns 12 as:

$$\frac{\partial \left(povsev\right)}{\partial \left(icts\right)}=-0.2923+\left(-5.1192\times \bar{fiacc}\right)$$

Where -0.2923 is the direct effect of ICT skills; -5.1192 coefficient of the interaction term for ICT skills and financial access; and $\bar{fiacc}$ is the average financial access score, which is 0.076 as apparent in Table 2.

$$\frac{\partial \left(povsev\right)}{\partial \left(icts\right)}=-0.2923+\left(-5.1192\times 0.076\right)=-0.927$$

Though both pathways are poverty severity-hindering, the finance access-ICT skill channel is more effective. This is plausible since as compared to financial development, financial access indicates the direct provision of resources to the private sector. Further, the result indicates that in the presence of financial inclusion, ICT skills can prove momentous in reducing the severity of poverty in SSA. Indeed, with tech-hubs springing up in the region, access to credit can aid the region's youthful population realise their innovative potentials. In a region where ICT skills are improving steadily, enhancing access to credit can prove crucial in transforming creative ideas into real income-generating business opportunities, which are essential for durable employment and poverty alleviation.

From our ancillary findings, there is evidence that both foreign aid and economic globalisation exert negative and statistically significant effects on the severity of poverty in SSA (Column 7). However, the effects are modest providing evidence for the less-inclusive sectors in which FDI, for instance, have been flowing into– the aviation, mining, and telecommunication sub-sectors (UNCTAD, 2019). Similar results are found for economic growth (Column 11) and social inclusion institutions (Column 9). The results signify the less-inclusive growth trajectories of the SSA in recent times, providing impetus for empirical contributions of this kind. Additionally, the results show that institutions for improving the ability, opportunities and dignities of the vulnerable can have a greater poverty severity reducing-effect if well resourced. The appropriateness of our system GMM estimates is evident in the AR (2) statistics showing the absence of second-order serial correlation in the residuals, and the Hansen P-values, providing evidence of the validity of our instruments.

### 4.4 Robustness check for poverty severity results

We check the robustness of our results in Table 3 using the Palma ratio as an outcome variable. The results as presented in Table 4 show that, except for economic globalisation, all our baseline covariates are statistically significant– vulnerable employment perpetuates poverty severity, while social inclusion institutions, foreign aid, and economic growth all suppress the severity of poverty in SSA.

**Table 3:** GMM results on the effects of financial development, financial access, and ICTs on the severity of poverty in SSA (Dependent variable: Squared Poverty Gap index).

Variable	(1)	(2)	(3)	(4)	(5)	(6)	(7)	(8)	(9)	(10)	(11)	(12)
Poverty severity (lag)	0.9871∗∗∗	0.9804∗∗∗	0.9861∗∗∗	1.0112∗∗∗	0.9425∗∗∗	1.0050∗∗∗	1.0115∗∗∗	0.9395∗∗∗	0.9852∗∗∗	1.0070∗∗∗	0.9188∗∗∗	0.9977∗∗∗
	(0.0043)	(0.0058)	(0.0079)	(0.0063)	(0.0098)	(0.0009)	(0.0102)	(0.0131)	(0.0041)	(0.0092)	(0.0153)	(0.0046)
Economic globalisation (KOF)	-0.0013∗∗∗	-0.0017∗∗	-0.0006	-0.0004	-0.0033∗∗∗	-0.0005	-0.0009	-0.0021	-0.0045∗∗∗	-0.0005	-0.0003	-0.0012∗∗
	(0.0002)	(0.0007)	(0.0005)	(0.0004)	(0.0011)	(0.0005)	(0.0007)	(0.0022)	(0.0010)	(0.0011)	(0.0019)	(0.0005)
Social inclusion	-0.0162∗∗∗	-0.0068	-0.0129∗	-0.0284∗∗∗	-0.0300∗∗	0.0033	-0.0361∗∗	-0.0526∗∗	-0.0323∗∗∗	-0.0466	-0.0251	0.0255
	(0.0041)	(0.0069)	(0.0071)	(0.0071)	(0.0126)	(0.0082)	(0.0136)	(0.0230)	(0.0088)	(0.0495)	(0.0260)	(0.0175)
Vulnerable employment	0.0005	0.0002	0.0006	0.0014∗∗∗	0.0020∗∗∗	0.0020∗∗∗	0.0029∗	-0.0028	0.0090∗∗∗	0.0014	0.0003	0.0013∗∗∗
	(0.0003)	(0.0007)	(0.0006)	(0.0004)	(0.0005)	(0.0004)	(0.0017)	(0.0028)	(0.0014)	(0.0015)	(0.0016)	(0.0004)
Foreign aid	-0.0001	-0.0003	-0.0003	-0.0008∗∗∗	-0.0003	-0.0012∗	-0.0005	-0.0007	-0.0014∗∗	-0.0009∗∗	-0.0011	-0.0032∗∗∗
	(0.0002)	(0.0003)	(0.0003)	(0.0003)	(0.0015)	(0.0006)	(0.0006)	(0.0025)	(0.0006)	(0.0004)	(0.0023)	(0.0007)
GDP growth	-0.0026∗∗∗	-0.0023∗∗∗	-0.0021∗∗∗	-0.0023∗∗∗	-0.0029	0.0004	-0.0024∗∗∗	0.0005	0.0008	-0.0021∗∗∗	-0.0026∗∗	-0.0003
	(0.0006)	(0.0004)	(0.0005)	(0.0005)	(0.0017)	(0.0003)	(0.0005)	(0.0016)	(0.0010)	(0.0007)	(0.0010)	(0.0004)
Financial development		-0.1046					-0.4085	-0.6981∗∗	-1.3712∗∗			
		(0.1164)					(0.3799)	(0.2974)	(0.5428)			
Financial access			-0.0465							-0.0926	-0.6331∗∗∗	-5.5405∗∗∗
			(0.1277)							(0.1727)	(0.1526)	(1.1142)
ICT access				-0.0048∗∗			-0.0090			-0.0023		
				(0.0021)			(0.0099)			(0.0054)		
ICT use					-0.0121∗∗∗			-0.0060			-0.0022	
					(0.0022)			(0.0053)			(0.0103)	
ICT skills						-0.1317∗∗∗			-0.2153∗∗∗			-0.2923∗∗∗
						(0.0256)			(0.0571)			(0.0525)
Financial development x ICT access							-0.0181					
							(0.0280)					
Financial development x ICT use								-0.0393				
								(0.0316)				
Financial development x ICT skills									-2.0551∗∗∗			
									(0.6161)			
Financial access x ICT access										-0.0098		
										(0.0124)		
Financial access x ICT use											-0.0194	
											(0.0333)	
Financial access x ICT skills												-5.1192∗∗∗
												(1.0173)
Constant	0.0894∗∗∗	0.1231	0.0509	-0.0443	0.1946∗∗∗	0.2233∗∗∗	-0.2021	0.6428∗∗	0.9376∗∗∗	0.0617	0.2498∗	0.2360∗∗∗
	(0.0259)	(0.0987)	(0.0705)	(0.0447)	(0.0646)	(0.0433)	(0.1812)	(0.2568)	(0.1462)	(0.1812)	(0.1474)	(0.0504)
Observations	1,636	1,636	1,636	1,636	608	913	1,636	608	913	1,636	608	913
Countries	42	42	42	42	41	42	42	41	42	42	41	42
Instruments	38	38	39	39	39	39	39	39	40	40	41	41
Wald $X^{2}$ statistic	283856	114100	121458	781405	132803	4.46100	303419	130891	796871	235612	113992	660383
Wald P-value	0.000	0.000	0.000	0.000	0.000	0.000	0.000	0.000	0.000	0.000	0.000	0.000
Net Effect	–	–	–	–	–	–	–	–	-0 .470	–	–	-0.927
Joint Significance Test (statistic)	–	–	–	–	–	–	–	–	11.13	–	–	25.32
Joint Significance Test P-value	–	–	–	–	–	–	–	–	0.0018	–	–	0.0000
Hansen P-Value	0.584	0.622	0.643	0.642	0.703	0.777	0.755	0.779	0.767	0.639	0.729	0.778
AR (1)	0.0003	0.0003	0.0003	0.0027	0.0143	0.0235	0.00279	0.0144	0.0226	0.00298	0.0148	0.0229
AR (2)	0.163	0.159	0.164	0.205	0.221	0.474	0.218	0.213	0.476	0.201	0.221	0.499

Standard errors in parentheses.

∗∗∗p < 0.01, ∗∗p < 0.05, ∗p < 0.1.

**Table 4:** GMM results on the effects of financial development, financial access, and ICTs on the severity of poverty in SSA (Dependent variable: Palma ratio).

Variable	(1)	(2)	(3)	(4)	(5)	(6)	(7)	(8)	(9)	(10)	(11)	(12)
Palma ratio (lag)	0.9245∗∗∗	0.9220∗∗∗	0.9273∗∗∗	0.9218∗∗∗	0.9881∗∗∗	0.7717∗∗∗	0.9198∗∗∗	0.9936∗∗∗	0.7647∗∗∗	0.9193∗∗∗	0.9863∗∗∗	0.7528∗∗∗
	(0.0009)	(0.0011)	(0.0009)	(0.0010)	(0.0006)	(0.0040)	(0.0011)	(0.0018)	(0.0051)	(0.0020)	(0.0007)	(0.0050)
Economic globalisation (KOF)	0.0002	-0.0013∗∗∗	0.0010∗∗∗	0.0002	0.0000	0.0025∗∗∗	-0.0012∗∗∗	-0.0034∗∗∗	0.0011	-0.0008	-0.0057∗∗∗	-0.0025
	(0.0001)	(0.0002)	(0.0004)	(0.0002)	(0.0006)	(0.0008)	(0.0004)	(0.0010)	(0.0012)	(0.0008)	(0.0012)	(0.0023)
Social inclusion	-0.0471∗∗∗	-0.0521∗∗∗	-0.0313∗∗	-0.0536∗∗∗	-0.1170∗∗∗	-0.1013∗∗∗	-0.0523∗∗∗	-0.0820∗∗	-0.1001	-0.0687∗∗∗	-0.0608	-0.1706∗
	(0.0097)	(0.0147)	(0.0145)	(0.0112)	(0.0277)	(0.0348)	(0.0166)	(0.0346)	(0.0643)	(0.0194)	(0.0436)	(0.0936)
Vulnerable employment	0.0019∗∗∗	0.0047∗∗∗	0.0022∗∗∗	0.0035∗∗∗	0.0026∗∗∗	0.0111∗∗∗	0.0062∗∗∗	0.0123∗∗∗	0.0171∗∗∗	0.0041∗∗∗	0.0040∗∗∗	0.0120∗∗∗
	(0.0002)	(0.0006)	(0.0003)	(0.0002)	(0.0004)	(0.0012)	(0.0011)	(0.0014)	(0.0018)	(0.0014)	(0.0007)	(0.0021)
Foreign aid	-0.0020∗∗∗	-0.0018∗∗∗	-0.0033∗∗∗	-0.0021∗∗∗	-0.0046∗∗∗	0.0087∗∗∗	-0.0016∗∗∗	-0.0019∗	-0.0098∗∗∗	-0.0028∗∗∗	-0.0041	-0.0129∗∗∗
	(0.0003)	(0.0003)	(0.0004)	(0.0004)	(0.0012)	(0.0009)	(0.0004)	(0.0011)	(0.0013)	(0.0006)	(0.0025)	(0.0017)
GDP growth	-0.0021∗∗∗	-0.0016∗	-0.0043∗∗∗	-0.0022∗∗∗	-0.0120∗∗∗	-0.0052∗∗∗	-0.0018∗∗	-0.0029∗∗∗	-0.0037∗	-0.0040∗∗∗	-0.0098∗∗∗	-0.0064∗∗
	(0.0006)	(0.0008)	(0.0004)	(0.0005)	(0.0011)	(0.0017)	(0.0007)	(0.0010)	(0.0019)	(0.0011)	(0.0017)	(0.0025)
Financial development		-0.3225∗∗∗					-0.3873	-2.0321∗∗∗	-3.6100∗∗∗			
		(0.0878)					(0.2976)	(0.1694)	(1.0947)			
Financial access			-0.0528∗∗∗							-0.0011	-0.0860∗∗∗	-0.1621∗∗∗
			(0.0068)							(0.0180)	(0.0188)	(0.0495)
ICT access				-0.0061∗∗∗			-0.0052			-0.0542∗∗∗		
				(0.0017)			(0.0048)			(0.0128)		
ICT use					-0.0267∗∗∗			-0.0075			-0.0039	
					(0.0061)			(0.0093)			(0.0038)	
ICT skills						-0.5415∗∗∗			-0.3568∗∗∗			-0.6869∗∗∗
						(0.0743)			(0.1259)			(0.1179)
Financial development x ICT access							-0.0035					
							(0.0194)					
Financial development x ICT use								-0.0574∗∗				
								(0.0214)				
Financial development x ICT skills									-4.1116∗∗∗			
									(1.0709)			
Financial access x ICT access										-0.1039∗∗∗		
										(0.0279)		
Financial access x ICT use											-0.0438∗∗∗	
											(0.0080)	
Financial access x ICT skills												-0.9208∗∗∗
												(0.3356)
Constant	0.5469∗∗∗	0.8482∗∗∗	0.4369∗∗∗	0.6736∗∗∗	-0.3261∗∗∗	2.9678∗∗∗	0.9838∗∗∗	1.1221∗∗∗	3.3274∗∗∗	0.7737∗∗∗	0.2503	4.2833∗∗∗
	(0.0298)	(0.0457)	(0.0608)	(0.0350)	(0.0922)	(0.1170)	(0.0906)	(0.1264)	(0.3184)	(0.0937)	(0.1647)	(0.4137)
Observations	1,638	1,638	1,492	1,638	610	915	1,638	610	915	1,492	599	853
Countries	42	42	42	42	41	42	42	41	42	42	41	41
Instruments	39	39	39	39	39	39	39	39	40	40	40	40
Wald $X^{2}$ statistic	154000	572900	153000	338000	697700	517770	236000	154400	120425	264000	315500	52547
Wald P-value	0.000	0.000	0.000	0.000	0.000	0.000	0.000	0.000	0.000	0.000	0.000	0.000
Net Effect	–	–	–	–	–	–	–	-0.0146	-0.8663	-0.6209	-0.0072	-0.7568
Joint Significance Test (statistic)	–	–	–	–	–	–	–	7.19	14.74	10.02	4.38	3.16
Joint Significance Test P-value	–	–	–	–	–	–	–	0.0106	0.000	0.0029	0.0427	0.0493
Hansen P-Value	0.642	0.573	0.667	0.586	0.563	0.861	0.593	0.756	0.858	0.678	0.666	0.802
AR (1)	0.0579	0.0578	0.0579	0.0578	0.0322	0.141	0.0579	0.0317	0.140	0.0578	0.0323	0.142
AR (2)	0.446	0.448	0.438	0.445	0.514	0.322	0.447	0.306	0.320	0.441	0.381	0.321

Standard errors in parentheses.

∗∗∗p < 0.01, ∗∗p < 0.05, ∗p < 0.

On our first objective, we find that the direct effects of financial development (Column 2), financial access (Column 3), and all the ICT indicators (Columns 4 – 6) are negative. Our results show that ICT skills and financial development are remarkable in reducing the gap between the rich and the poor in terms of income growth. Our results thus corroborate that of Appiah Otoo and Song (2021). For the second objective, we find that all our ICT dynamics and financial development pathways are negative and statistically significant. As presented in Table 3, we find that the financial development-ICT skills (Column 9) and financial access-ICT skills (Column 12) pathways are the most relevant complementary channels for reducing the severity of poverty in SSA. In specifics, we find that the net effects of 1 per cent improvement in ICT skills in line with the financial development and financial access are –0.87 per cent and –0.76 per cent, respectively. Likewise, we find that enhancing ICT usage by 1 per cent given current levels of financial development and financial access reduces the severity of poverty in SSA by 0.01 (Column 7) and 0.007 (Column 11), respectively. All the joint significance tests are also significant, signifying the need for policymakers interested in Africa's development agenda to broaden or support the private sector in enhancing ICT access and ICT usage in the region. Indeed, these avenues provide direct opportunities for the masses who can deal directly in ICT businesses, be it retail, repairs, or innovation. The results provide some form of optimism through the use of ICTs, which in itself boost financial inclusion, for creating opportunities, and reducing inequality among households. Further, the pathway results indicate that in addressing the welfare setbacks due to COVID-19, for instance, the youth-friendly channel of ICT can be harnessed in line with greater financial deepening to reduce the severity of poverty in SSA. The results also indicate that the lack of contemporary assets like ICTs amplifies the severity of poverty in settings like the SSA where social protection is lacking (Lustig et al., 2019). Albeit modest effects, our controls– economic growth, foreign aid, and social inclusion also exert negative effects on the severity of poverty in SSA.

### 4.5 System GMM results on the effects of financial development and ICTs on the intensity of poverty in SSA

We shift focus to the results on the effects of ICTs, financial development and financial access on the intensity of poverty in SSA (see Table 5). We find empirical evidence from our baseline estimates to show that economic growth, foreign aid, and social inclusion are significant in reducing the intensity of poverty in SSA. These results are based on Eq. (3).

**Table 5:** GMM results on the effects of financial development, financial access, and ICTs on the intensity of poverty in SSA (Dependent variable: Poverty Gap (US$1.90)).

Variable	(1)	(2)	(3)	(4)	(5)	(6)	(7)	(8)	(9)	(10)	(11)	(12)
Poverty intensity (lag)	0.9823***	0.9123***	0.9663***	0.9464***	0.9098***	1.0132***	0.9044***	0.9077***	1.0043***	0.8866***	0.9135***	1.0021***
	(0.0068)	(0.0222)	(0.0082)	(0.0067)	(0.0100)	(0.0038)	(0.0198)	(0.0218)	(0.0106)	(0.0207)	(0.0093)	(0.0037)
Economic globalisation (KOF)	-0.0013***	-0.0060***	-0.0008**	-0.0013***	-0.0022***	-0.0004	-0.0030***	-0.0019	-0.0010***	-0.0000	-0.0005	-0.0007***
	(0.0003)	(0.0009)	(0.0003)	(0.0004)	(0.0006)	(0.0002)	(0.0007)	(0.0017)	(0.0003)	(0.0005)	(0.0013)	(0.0002)
Social inclusion	-0.0065*	-0.0094	-0.0019	-0.0001	0.0103	0.0013	0.0149	-0.0029	-0.0094	0.0122	-0.0194***	-0.0076*
	(0.0035)	(0.0227)	(0.0039)	(0.0072)	(0.0127)	(0.0049)	(0.0240)	(0.0150)	(0.0060)	(0.0160)	(0.0072)	(0.0040)
Vulnerable employment	0.0001	0.0061***	0.0005***	0.0010**	0.0006*	0.0014***	0.0048***	0.0018*	0.0038***	0.0032***	0.0004	0.0013***
	(0.0002)	(0.0015)	(0.0002)	(0.0005)	(0.0004)	(0.0002)	(0.0013)	(0.0009)	(0.0004)	(0.0009)	(0.0003)	(0.0002)
Foreign aid	-0.0003**	-0.0014***	-0.0003	-0.0005*	-0.0030***	-0.0008***	-0.0013***	-0.0014	-0.0008***	-0.0018***	-0.0025***	-0.0010***
	(0.0001)	(0.0002)	(0.0002)	(0.0003)	(0.0008)	(0.0002)	(0.0003)	(0.0010)	(0.0003)	(0.0004)	(0.0009)	(0.0002)
GDP growth	-0.0008***	-0.0003	-0.0013***	-0.0012***	-0.0033***	-0.0002	-0.0015**	-0.0015	-0.0001	-0.0005	-0.0019*	-0.0002
	(0.0003)	(0.0005)	(0.0003)	(0.0003)	(0.0006)	(0.0001)	(0.0007)	(0.0013)	(0.0003)	(0.0006)	(0.0010)	(0.0002)
Financial development		-1.0548***					-0.6120***	-0.3753***	-0.3934			
		(0.1990)					(0.1511)	(0.1186)	(0.3574)			
Financial access			-0.0251***							-0.0658***	-0.0267***	-0.0234***
			(0.0049)							(0.0117)	(0.0098)	(0.0048)
ICT (access)				-0.0091***			-0.0199***			-0.0132**		
				(0.0019)			(0.0060)			(0.0049)		
ICT (use)					-0.0153***			-0.0058**			-0.0095	
					(0.0016)			(0.0023)			(0.0112)	
ICT (skills)						-0.0736***			-0.1117**			-0.0698***
						(0.0130)			(0.0451)			(0.0140)
Financial development x ICT (access)							-0.0193**					
							(0.0078)					
Financial development x ICT (use)								-0.0320				
								(0.0197)				
Financial development x ICT (skills)									-0.6794*			
									(0.3431)			
Financial access x ICT (access)										-0.0062		
										(0.0134)		
Financial access x ICT (use)											-0.0592**	
											(0.0275)	
Financial access x ICT (skills)												-0.1308***
												(0.0286)
Constant	0.1132***	1.0517***	0.0613**	0.2788***	0.2405***	0.1184***	0.7647***	0.5005***	0.3616***	0.2775***	0.1849**	0.0516
	(0.0212)	(0.1349)	(0.0293)	(0.0484)	(0.0507)	(0.0182)	(0.1141)	(0.1614)	(0.0963)	(0.0642)	(0.0906)	(0.0335)
Observations	1,636	1,636	1,490	1,636	608	913	1,636	608	913	1,490	597	851
Countries	42	42	42	42	41	42	42	41	42	42	41	41
Instruments	39	39	39	39	41	40	40	40	40	40	41	40
Wald $X^{2}$ statistic	520500	22581	215946	594835	817925	50500	29971	491752	183100	29586	48790	18400
Wald P-value	0.000	0.000	0.000	0.000	0.000	0.000	0.000	0.000	0.000	0.000	0.000	0.000
Net Effect	–	–	–	–	–	–	-0.0222	–	-0.1959	–	-0.0139	-0.0797
Joint Significance Test (statistic)	–	–	–	–	–	–	6.07	–	3.92	–	4.50	3.11
Joint Significance Test P-value	–	–	–	–	–	–	0.018	–	0.0544	–	0.0400	0.0804
Hansen P-Value	0.718	0.590	0.629	0.685	0.832	0.838	0.667	0.840	0.724	0.570	0.856	0.687
AR (1)	0.0002	0.0001	0.0004	0.0026	0.0117	0.0334	0.0024	0.0120	0.0320	0.0033	0.0129	0.0334
AR (2)	0.233	0.186	0.276	0.290	0.275	0.416	0.255	0.267	0.397	0.251	0.268	0.443

Standard errors in parentheses.

***p < 0.01, **p < 0.05, *p < 0.1.

Regarding our first hypothesis, we find strong evidence to show that all our key variables (i.e., ICT access, ICT skills, ICT usage, financial development and financial access) directly suppress the intensity of poverty in SSA. Particularly, the development of the region's financial sector reduces the intensity of poverty by 1 per cent (Column 2). Likewise, enhancing financial access by 1 per cent in SSA has the potency of reducing the intensity of poverty by 0.03 per cent (Column 3). The results further unveil that enhancing the region's ICT access, ICT usage, and ICT skills can reduce the intensity of poverty by 0.01 per cent, 0.02 per cent, and 0.07 per cent, respectively (see Columns 4 – 6).

The results as apparent in Table 5 further show that all the ICT-finance interaction terms are negative, providing evidence for our second objective. The uniqueness of our results is that all our ICT indicators form synergies with finance in reducing the intensity of poverty in SSA. For instance, we find strong empirical evidence that given the current efficiency, depth, and access of SSA's financial sector, every 1 per cent improvement in ICT access and ICT skills reduces the intensity of poverty by 0.02 per cent and 0.19 per cent, respectively. Similar results are found for both the financial access-ICT usage, and financial access-ICT skills pathways. We report a net effect of -0.08 per cent for the latter and -0.01 per cent for the former. The results suggest that ICT diffusion can thus be targeted to improve people's livelihoods, achieve gender equality in labour force participation, and poverty reduction in SSA^19^. Further, in settings where inequality in assets and capital distribution perpetuate poverty (Fosu, 2015), the ICT diffusion can be harnessed in line with enhanced financial access to promote human and socioeconomic development (Ofori and Asongu, 2021b; Andrés et al., 2017). This is more so as there is a high prospect and growing ecosystem for ICT penetration and innovation, whose economic impacts can reverberate throughout the region resulting in a better livelihood for the masses.

From our auxiliary findings, we find that vulnerable employment induces the intensity of poverty in SSA. This is in line with the result of Ofori (2021) and Ofori et al. (2021) who provide robust evidence to show that vulnerable employment hampers inclusive growth. Institutions for social inclusion, economic growth, foreign aid, and economic globalisation, however, prove significant in reducing the intensity of poverty in SSA (Column 12). Our results thus indicate that strategic investment in the AfCFTA can boost growth, create opportunities, and reduce the intensity of poverty in SSA. The significant but modest effect of social inclusion indicates a greater potential of reducing the intensity of poverty through policies that aim at levelling the playing field in the form of fair redistribution and inclusion. This is particularly imperative considering the reversal of welfare gains due to the COVID-19 pandemic.

### 4.6 Robustness check for poverty intensity results

We evaluate the robustness of our results on the intensity of poverty using the lower middle-income poverty gap of US$3.20 as a new outcome variable. The results are provided in Table 6. For our first hypothesis, we find that the direct effects of financial development, financial access, ICT access, ICT usage and ICT skills are all negative and statistically significant. For instance, enhancing financial access by 1 per cent reduces the intensity of poverty by 0.03 per cent (Column 3). As we find in the main results in Table 5, ICT skills ranks high (0.1%) compared to the other components such as ICT usage (0.005%) and access (0.01%).

**Table 6:** GMM results on the effects of financial development, financial access, and ICTs on the intensity of poverty in Sub-Saharan Africa (Dependent variable: Poverty Gap (US$3.20)).

Variable	(1)	(2)	(3)	(4)	(5)	(6)	(7)	(8)	(9)	(10)	(11)	(12)
Poverty intensity (lag)	0.9555∗∗∗	0.9220∗∗∗	0.9434∗∗∗	0.9152∗∗∗	0.8930∗∗∗	0.9777∗∗∗	0.9227∗∗∗	0.9493∗∗∗	0.9658∗∗∗	0.9158∗∗∗	0.8887∗∗∗	0.9388∗∗∗
	(0.0065)	(0.0101)	(0.0117)	(0.0077)	(0.0077)	(0.0038)	(0.0119)	(0.0068)	(0.0037)	(0.0158)	(0.0203)	(0.0074)
Economic globalisation (KOF)	-0.0003∗∗∗	-0.0019∗∗∗	-0.0003∗	-0.0008∗∗∗	-0.0011∗	0.0003	-0.0010∗∗∗	-0.0018∗∗	-0.0002	-0.0007	-0.0008	-0.0007∗∗
	(0.0001)	(0.0003)	(0.0002)	(0.0002)	(0.0006)	(0.0002)	(0.0004)	(0.0008)	(0.0002)	(0.0004)	(0.0008)	(0.0004)
Social inclusion	-0.0023	-0.0150∗	-0.0107∗∗	-0.0103	-0.0005	-0.0152∗∗∗	-0.0188∗∗∗	-0.0107	-0.0156∗∗∗	-0.0300∗∗∗	-0.0499∗∗∗	-0.0242∗∗∗
	(0.0022)	(0.0089)	(0.0048)	(0.0062)	(0.0075)	(0.0039)	(0.0062)	(0.0099)	(0.0055)	(0.0108)	(0.0134)	(0.0064)
Vulnerable employment	0.0004∗∗∗	0.0015∗∗∗	0.0004∗∗	0.0008∗∗∗	0.0015∗∗∗	0.0008∗∗∗	0.0016∗∗∗	0.0006	0.0019∗∗∗	0.0031∗∗∗	0.0010∗∗	0.0015∗∗∗
	(0.0001)	(0.0003)	(0.0002)	(0.0003)	(0.0004)	(0.0001)	(0.0004)	(0.0004)	(0.0003)	(0.0005)	(0.0004)	(0.0002)
Foreign aid	-0.0004∗∗∗	-0.0007∗∗∗	-0.0006∗∗∗	-0.0007∗∗∗	-0.0008∗∗∗	-0.0002	-0.0006∗∗∗	-0.0002	-0.0004∗∗	-0.0009∗∗∗	-0.0012∗	-0.0005
	(0.0001)	(0.0001)	(0.0002)	(0.0001)	(0.0003)	(0.0001)	(0.0002)	(0.0004)	(0.0002)	(0.0003)	(0.0007)	(0.0004)
GDP growth	-0.0011∗∗∗	-0.0013∗∗∗	-0.0008∗∗∗	-0.0013∗∗∗	-0.0012∗	-0.0001	-0.0010∗∗∗	-0.0014∗∗	-0.0002	-0.0005	-0.0023∗∗∗	-0.0001
	(0.0002)	(0.0003)	(0.0002)	(0.0003)	(0.0007)	(0.0001)	(0.0003)	(0.0005)	(0.0002)	(0.0004)	(0.0008)	(0.0003)
Financial development		-0.3116∗∗∗					-0.4652∗∗∗	-0.2705∗∗∗	-0.5696∗∗			
		(0.0491)					(0.0719)	(0.0725)	(0.2466)			
Financial access			-0.0339∗∗∗							-0.0458∗∗∗	-0.0722∗∗∗	-0.0318∗∗∗
			(0.0053)							(0.0109)	(0.0124)	(0.0056)
ICT access				-0.0099∗∗∗			-0.0237∗∗∗			-0.0228∗∗∗		
				(0.0009)			(0.0021)			(0.0029)		
ICT use					-0.0049∗			-0.0190∗∗∗			-0.0200	
					(0.0027)			(0.0031)			(0.0176)	
ICT skills						-0.0981∗∗∗			-0.0730∗∗			-0.0964∗∗∗
						(0.0111)			(0.0339)			(0.0233)
Financial development x ICT access							-0.0617∗∗∗					
							(0.0020)					
Financial development x ICT use								-0.1074∗∗∗				
								(0.0098)				
Financial development x ICT skills									-0.7056∗∗∗			
									(0.2496)			
Financial access x ICT access										-0.0310∗∗∗		
										(0.0076)		
Financial access x ICT use											-0.0161	
											(0.0531)	
Financial access x ICT skills												-0.0899∗∗
												(0.0423)
Constant	0.1291∗∗∗	0.4319∗∗∗	0.0592∗	0.3567∗∗∗	0.2897∗∗∗	0.1419∗∗∗	0.4269∗∗∗	0.2923∗∗∗	0.2607∗∗∗	0.3097∗∗∗	0.1106	0.1711∗∗∗
	(0.0206)	(0.0457)	(0.0305)	(0.0340)	(0.0478)	(0.0167)	(0.0525)	(0.0631)	(0.0392)	(0.0554)	(0.0917)	(0.0369)
Observations	1,638	1,638	1,492	1,638	610	915	1,638	610	915	1,492	599	853
Countries	42	42	42	42	41	42	42	41	42	42	41	41
Instruments	38	39	39	39	39	39	39	39	40	40	40	40
Wald $X^{2}$ statistic	401900	289821	120400	1589000	231000	771000	140918	256300	333000	102200	855698	347300
Wald P-value	0.000	0.000	0.000	0.000	0.000	0.000	0.000	0.000	0.000	0.000	0.000	0.000
Net Effect	–	–	–	–	–	–	-0.0313	-0.0323	-0.0947	-0.025i	–	-0.1032
Joint Significance Test (statistic)	–	–	–	–	–	–	16.10	7.19	14.74	10.02		4.38
Joint Significance Test P-value	–	–	–	–	–	–	0.0003	0.0106	0.0004	0.0029		0.0427
Hansen P-Value	0.726	0.626	0.633	0.714	0.756	0.930	0.742	0.833	0.849	0.764	0.688	0.865
AR (1)	0.0274	0.027	0.003	0.049	0.065	0.089	0.041	0.053	0.091	0.011	0.022	0.032
AR (2)	0.165	0.152	0.333	0.178	0.154	0.568	0.174	0.147	0.582	0.300	0.228	0.339

Standard errors in parentheses.

∗∗∗p < 0.01, ∗∗p < 0.05, ∗p < 0.1.

We find evidence for our second hypothesis as well. We find that ICT and finance can be effective channels for reducing the intensity of poverty in SSA. From the financial development–ICT channels, we find that enhancing ICT access, ICT usage and ICT skills by 1 per cent given the current state of the region's financial development reduces the intensity of poverty by 0.03 per cent, 0.03 per cent and 0.09 per cent, respectively. The results further show that while enhancing ICT usage, ICT skills and ICT access can reduce the intensity of poverty in SSA, the effect can be amplified with enhanced financial access. As we found in the case of the severity of poverty in SSA, ICT skills are key, both conditionally or unconditionally in reducing the intensity of poverty in SSA.

Results from the PCSE as apparent in the supplementary material (i.e., Tables A.4 – A.7) show that our variables of interest are indeed relevant in addressing the welfare issues of poverty intensity and severity.

## 5 Conclusion and policy recommendations

Motivated by the need to address the bleak socioeconomic outlook of SSA in the wake of the COVID-19 and offer suggestions towards the region's efforts in reducing global extreme poverty below 7 per cent by 2030, we explore how ICTs, financial development, and financial access can be targeted to reduce the severity and intensity of poverty in SSA. To this end, we draw on data for the period 1980–2019 on 42 countries for the analysis.

We provide evidence robust to several specifications from the dynamic system GMM that although unconditionally, ICTs reduce the severity and intensity of poverty in SSA, the effects are pronounced in the presence of financial development and financial access. Considering the fact that challenges arising from poverty and inequality among households have material and non-material (information, communication or knowledge) elements, investing in ICTs in the presence of a dynamic, efficient and innovative financial sector can be a gamechanger in SSA's shared growth pursuits. A key finding from the result is that, among all the components of ICT diffusion, it is ICT skills that form remarkable synergies with financial development and financial access in reducing both the severity and intensity of poverty in SSA.

We conclude, therefore, that ICTs and finance are effective channels that can be employed by decisionmakers in SSA to improve livelihood outcomes in terms of improvement in people's material or non-material lives. We thus affirm our hypotheses. For our ancillary findings, we conclude that while economic growth and globalisation matter most for addressing both the severity and intensity of poverty, social inclusion policies matter only for addressing the former whereas foreign aid is crucial for addressing the latter. This can prove crucial in addressing the marked poverty, inequality, unemployment and social tensions in the region. Considering challenges in raising development finance and the deep-rooted nature of poverty in SSA, fighting the socioeconomic problem may not be about enhancing infrastructural investment per se but infrastructural development of opportunities and inclusiveness. Aside from the remarkable poverty severity and intensity eradication effects of ICT skills, ICT usage, ICT access and financial deepening, is the added benefit of reducing human resource wastage, the enhancement of knowledge and skills, and increased capacity to prepare and/or deal with shocks.

The attendant recommendations for policy considerations are as follows. First, we recommend that African leaders prioritise the development of ICT skills, ICT access and ICT usage. The long-term benefit of this will be the creation of decent jobs, improved financial inclusion, an effective fight against climate change, and tax evasion. This can be enhanced if development partners such as the African Development Bank, the International Monetary Fund and the World Bank channel technical, monetary and logistical support to complement various governments efforts towards the broadening/deepening of ICT access, ICT skills and ICT usage especially in the hinterlands where gaps in these assets are marked. Further, for African leaders to realise the relevance of ICTs in addressing the severity and intensity of poverty, lubricating mechanisms such as the development of the region's tech-hubs should be pursued. This can reduce deprivation by providing the region's youthful population high-tech ideas commercialisation, patent development and start-up company incubation to offer technical and logistical support to take advantage of the opportunities such as the one provided by the AfCFTA to reduce poverty. Finally, efforts to enhance financial access and social inclusion should be a priority to cushion the private sector build capacity, address human resource wastage and contribute to national development. For the academic community, similar contributions could be made by exploring whether the synergies we find for ICTs and financial development, and financial access matter for income inequality as well. Finally, this study can be replicated at the sub-regional level such as in West Africa, North Africa, and Eastern and Southern Africa to inform regional policy discourses on efforts aimed at addressing poverty severity and intensity.

The first drawback to this study is that we do not explore the effects of financial market access on the intensity and severity of poverty since the region's financial market is generally underdeveloped. Second, countries such as Eritrea, Somalia, South Sudan and Zimbabwe are not considered due to limited data. With data availability and a well-developed financial market, future works can draw on the arguments espoused in this study to test our hypotheses.

## Declarations

### Author contribution statement

Isaac Kwesi Ofori: Conceived and designed the experiments; Performed the experiments; Analyzed and interpreted the data; Contributed reagents, materials, analysis tools or data; Wrote the paper.

Francis Taale: Conceived and designed the experiments; Contributed reagents, materials, analysis tools or data; Wrote the paper.

Mark Kojo Armah: Conceived and designed the experiments; Contributed reagents, materials, analysis tools or data; Wrote the paper.

Pamela Efua Ofori: Analyzed and interpreted the data; Contributed reagents, materials, analysis tools or data; Wrote the paper.

### Funding statement

This research did not receive any specific grant from funding agencies in the public, commercial, or not-for-profit sectors.

### Data availability statement

Data will be made available on request.

### Declaration of interests statement

The authors declare no conflict of interest.

### Additional information

No additional information is available for this paper.

## Figures and Tables

**Table A.1:** Pairwise correlations.

	(1)	(2)	(3)	(4)	(5)	(6)	(7)	(8)	(9)	(10)	(11)	(12)	(13)	(14)
(1) Poverty severity	1.000													
(2) Poverty gap (US$3.20)	0.822	1.000												
(3) Economic globalisation	-0.290	-0.409	1.000											
(4) Social inclusion	-0.040	0.059	0.053	1.000										
(5) Vulnerable employment	0.327	0.512	-0.454	0.046	1.000									
(6) Foreign aid	-0.133	-0.229	-0.109	0.102	0.184	1.000								
(7) GDP growth	-0.073	-0.049	0.112	0.173	-0.016	-0.021	1.000							
(8) Financial development	-0.305	-0.407	0.475	-0.051	-0.513	-0.320	0.028	1.000						
(9) Financial access	-0.301	-0.542	0.560	0.033	-0.381	-0.161	0.026	0.674	1.000					
(10) ICT (skills)	-0.398	-0.493	0.558	0.208	-0.668	-0.260	0.101	0.452	0.487	1.000				
(11) ICT (access)	-0.252	-0.506	0.536	-0.009	-0.436	-0.194	0.027	0.611	0.775	0.415	1.000			
(12) ICT (use)	-0.077	-0.309	0.470	-0.034	-0.220	-0.114	-0.047	0.388	0.568	0.253	0.737	1.000		
(13) Palma ratio	0.136	0.103	-0.045	0.063	-0.077	0.045	-0.026	0.039	-0.021	-0.084	-0.029	0.043	1.000	
(14) Poverty intensity	0.941	0.956	-0.343	0.005	0.429	0.187	-0.068	-0.361	-0.435	-0.457	-0.388	-0.200	0.148	1.000

**Table A.2:** Unit root test results.

Variables	Constant		1^st^ Difference	
	CIPS (W-t-bar)	CADF	CIPS (W-t-bar)	CADF
*Poverty severity*	5.852	79.229∗∗∗	-13.292∗∗∗	-1.853
*Palma ratio*	2.778	99.627∗∗∗	-19.627∗∗∗	-0.928
Poverty intensity	6.085	51.235∗∗∗	-12.906∗∗∗	0.139
Poverty gap (US$3.20)	6.679	67.611∗∗∗	-13.563∗∗∗	2.799∗∗
Vulnerable employment	142.294∗∗∗	-2.363∗∗	18.824∗∗∗	-2.771∗∗
Social inclusion	4.092	60.476∗∗∗	-6.089∗∗∗	7.169∗∗∗
Foreign aid	-1.637∗	63.885∗∗∗	-24.141∗∗∗	-4.266
GDP growth	-16.789∗∗∗	8.528∗∗∗	-38.909∗∗∗	-6.043
Economic globalisation	0.670	58.559∗∗∗	-17.782∗∗∗	1.363∗
Financial development	-1.699∗∗	62.487∗∗∗	-15.918∗∗∗	2.534∗∗
Financial access	-0.710	50.221∗∗∗	-5.483∗∗∗	3.083∗∗
ICT usage	-1.281	61.026∗∗∗	-10.120∗∗∗	5.829∗∗∗
ICT access	11.046	31.506∗∗∗	1.872∗∗∗	-5.834
ICT skills	0.079	51.235∗∗∗	-15.514∗∗∗	0.139

Note: ∗∗∗ p<0.01, ∗∗ p<0.05, ∗ p<0.1; CIPS refers to Cross-sectionally Augmented Im Pesaran Shin; CADF means Cross-sectionally Augmented Dickey–Fuller.

Both CADF & CIPS test the H_0_: All panels contain unit root against H_1_: Some panels are stationary.

**Table A.3:** Bivariate results on the effects of ICTs, financial access, and financial development on the severity and intensity of poverty in SSA. Dependent Variable: Squared poverty gap index Dependent Variable: Poverty intensity.

Variable	(1)	(2)	(3)	(4)	(5)	(6)	(7)	(8)	(9)	(10)
Financial development	-11.6915∗∗∗					-6.6505∗∗∗				
	(0.5829)					(0.3150)				
Financial access		-12.1655∗∗∗					-6.5720∗∗∗			
		(0.3367)					(0.1852)			
ICT (access)			-0.3237∗∗∗					-0.1770∗∗∗		
			(0.0092)					(0.0050)		
ICT (use)				-0.4062∗∗∗					-0.2190∗∗∗	
				(0.0313)					(0.0174)	
ICT (skills)					-4.2137∗∗∗					-2.2284∗∗∗
					(0.2850)					(0.1585)
Constant	2.9781∗∗∗	2.4599∗∗∗	2.2267∗∗∗	1.3274∗∗∗	4.6363∗∗∗	3.4982∗∗∗	3.1768∗∗∗	3.0548∗∗∗	2.5304∗∗∗	4.3115∗∗∗
	(0.0887)	(0.0502)	(0.0482)	(0.0896)	(0.2333)	(0.0479)	(0.0276)	(0.0263)	(0.0498)	(0.1297)
Observations	1,680	1,680	1,492	1,492	1,680	1,680	1,680	1,492	1,492	1,680
R-squared	0.1897	0.4317	0.4274	0.2176	0.1890	0.2059	0.4227	0.4291	0.2076	0.1741
Adjusted R-Squared	0.189	0.431	0.427	0.216	0.188	0.205	0.422	0.429	0.206	0.173

Standard errors in parentheses.

∗∗∗ p<0.01, ∗∗ p<0.05, ∗ p<0.1.

## Footnotes

1 The Africa Agenda 2063 is long-term goal that shows the resolve on the part of African leaders to build quality institutions, foster durable shared growth trajectories, reduce aid dependency, improve the quality of life and deepen Africa's voice and competitiveness at the International level (Africa Union, 2015).

2 In respective terms, the SDGs 1, 8 and 10 seek to end poverty, foster descent work and economic growth, and ensure equitable income distribution (i.e., reduce income inequality).

3 Compared to other developing regions, Brown et al. (2020) highlights the virtual non-existence of home environment protection in the SSA.

4 Indeed, changes in weather patterns have been realised, with massive rainfall in East Africa, and low rainfall in West Africa causing food production challenges.

5 Socio-political tensions have deepened in the SSA in recent times, particularly, in Nigeria, Somalia, Mali, Cameroon, and Niger.

6 Green growth is needed to among others address human capital/resources wastage, enhance innovation and green-collar jobs, address climate change, protect biodiversity and water resources while presenting the regions with the surest way of lessening the impacts of future socioeconomic shocks.

7 The severity of poverty captures inequalities or differences in income levels among poor households while the intensity of poverty captures the average deprivation of each household.

8 Sen argues that matters in people's well-being is what they are capable of being or doing with the goods to which they have access.

9 Examples are mobile phones, tablets, computers, internet, radios, televisions, audio visuals, printers, and related software for application in several facets of life.

10 ICT thus consolidates financial allocation efficiency of financial institutions through cost reduction, and the optimal channelling of resources from savers to investors.

11 Such is the example of the Asia Pacific region, where countries such as Hong Kong, Taiwan, Singapore, and South Korea leapfrogged development through ICT.

12 Major tech-hubs in SSA are the SmartXchange, RLABS, and JoziHub of South Africa; Kinu of Tanzania; iSPACE, and Meltwater Entrepreneurial School of technology Hub of Ghana; xHub, IHub, Swahili Box, eMOBILIS, and Afrinovator of Kenya; and Co-creation Hub, Wennovation Hub, Focus Hub of Nigeria.

13 Angola, Benin, Botswana, Burkina Faso, Burundi, Cabo Verde, Cameroon, Central African Republic, Chad, Comoros, Congo, DR., Congo, Cote d'Ivoire, Ethiopia, Gabon, The Gambia, Guinea, Ghana, Guinea Bissau, Kenya, Lesotho, Liberia, Madagascar, Malawi, Mali, Mauritania, Mauritius, Mozambique, Namibia, Niger, Nigeria, Rwanda, Sao Tome and Principe, Senegal, Seychelles, Sierra Leone, Sudan, South Africa, Tanzania, Togo, Uganda, Zambia.

14 The poverty gap US$1.90 a day (2011 PPP) is the mean shortfall in income or consumption from the poverty line of $1.90 a day (counting the nonpoor as having zero shortfall), expressed as a percentage of the poverty line.

15 Lack of such asset in themselves is an indicator of poverty (Castells, 1999).

16 The KOF globalization index measures the degree of globalization of 122 countries. The index provides statistics on three main dimensions of interaction– economic, social, and political.

17 In all GMM estimations, the instruments used are the lags of the regressors. The appropriateness of the estimates is evaluated based on the test for validity of the instruments, the Wald test, and the serial correlation test of the residuals.

18 The graphs and the empirical results are generated using the STATA (v.16.0) statistical software.

19 In part, these results are an empirical response to Asongu (2013), who suggested that such an adventure can be undertaken given the link between financial development and ICTs diffusion in Africa.
