# Supplementary material for: Addressing the severity and intensity of poverty in Sub-Saharan Africa: how relevant is the ICT and financial development pathway?
Source: Heliyon. 2021 Oct 12;7(10):e08156. doi: 10.1016/j.heliyon.2021.e08156 (PMC8546426; doi:10.1016/j.heliyon.2021.e08156)
Supplement: Supplementary file 1 — Supplementary-file [file mmc1.docx]

**Supplementary Results (Tables A.3 – A.6)**

**Results Are Based on The Panel Corrected Standard Errors Estimation (PCSE)Technique**

***Table A.4: PCSE results on the effects of financial development, financial access, and ICTs on the severity of poverty in SSA (Dependent variable: Squared poverty gap index)***

| Variables | (1) | (2) | (3) | (4) | (5) | (6) | (7) | (8) | (9) | (10) | (11) | (12) |
| --- | --- | --- | --- | --- | --- | --- | --- | --- | --- | --- | --- | --- |
| **Squared poverty gap index** (lag) | 0.9631*** | 0.9616*** | 0.9411*** | 0.9689*** | 0.9487*** | 0.9577*** | 0.9689*** | 0.9496*** | 0.9249*** | 0.9662*** | 0.9405*** | 0.9251*** |
|  | (0.0135) | (0.0135) | (0.0131) | (0.0078) | (0.0277) | (0.0206) | (0.0077) | (0.0284) | (0.0222) | (0.0086) | (0.0331) | (0.0227) |
| Economic globalisation (KOF) | -0.0064*** | -0.0056*** | -0.0031** | -0.0017* | -0.0049* | -0.0074*** | -0.0017* | -0.0036 | -0.0037** | -0.0014 | -0.0027 | -0.0035** |
|  | (0.0014) | (0.0013) | (0.0012) | (0.0009) | (0.0028) | (0.0019) | (0.0009) | (0.0031) | (0.0017) | (0.0010) | (0.0030) | (0.0017) |
| Social inclusion | 0.0137 | 0.0115 | 0.0257 | -0.0115 | -0.0275 | 0.0047 | -0.0116 | -0.0371 | 0.0233 | -0.0068 | -0.0228 | 0.0238 |
|  | (0.0223) | (0.0221) | (0.0220) | (0.0215) | (0.0565) | (0.0244) | (0.0216) | (0.0557) | (0.0254) | (0.0216) | (0.0564) | (0.0259) |
| Vulnerable employment | -0.0007* | -0.0011** | -0.0005 | -0.0003 | -0.0002 | -0.0001 | -0.0003 | -0.0006 | 0.0008 | -0.0006 | -0.0007 | 0.0007 |
|  | (0.0004) | (0.0005) | (0.0004) | (0.0004) | (0.0009) | (0.0008) | (0.0004) | (0.0009) | (0.0008) | (0.0004) | (0.0009) | (0.0009) |
| Foreign aid | 0.0004 | -0.0002 | 0.0001 | 0.0001 | 0.0001 | 0.0001 | 0.0001 | -0.0004 | -0.0000 | 0.0001 | 0.0002 | -0.0002 |
|  | (0.0013) | (0.0014) | (0.0013) | (0.0009) | (0.0037) | (0.0016) | (0.0010) | (0.0039) | (0.0016) | (0.0009) | (0.0037) | (0.0017) |
| GDP growth | -0.0008 | -0.0009 | -0.0014 | -0.0027 | -0.0036 | -0.0008 | -0.0027 | -0.0036 | -0.0017 | -0.0026 | -0.0043 | -0.0017 |
|  | (0.0026) | (0.0026) | (0.0025) | (0.0017) | (0.0076) | (0.0033) | (0.0017) | (0.0075) | (0.0032) | (0.0017) | (0.0075) | (0.0032) |
| Financial development |  | -0.3802** |  |  |  |  | 0.0115 | -0.2338 | 0.1986 |  |  |  |
|  |  | (0.1568) |  |  |  |  | (0.1613) | (0.4155) | (0.3437) |  |  |  |
| Financial access |  |  | -0.7886** |  |  |  |  |  |  | -0.2684** | -0.5097 | 1.0042 |
|  |  |  | (0.3389) |  |  |  |  |  |  | (0.1264) | (0.3811) | (1.7721) |
| ICT access |  |  |  | -0.0089*** |  |  | -0.0086** |  |  | -0.0110*** |  |  |
|  |  |  |  | (0.0028) |  |  | (0.0042) |  |  | (0.0038) |  |  |
| ICT use |  |  |  |  | -0.0118 |  |  | -0.0039 |  |  | -0.0095 |  |
|  |  |  |  |  | (0.0119) |  |  | (0.0078) |  |  | (0.0095) |  |
| ICT skills |  |  |  |  |  | -0.0337 |  |  | -0.0399 |  |  | -0.0263 |
|  |  |  |  |  |  | (0.0317) |  |  | (0.0320) |  |  | (0.0222) |
| Financial development x ICT access |  |  |  |  |  |  | -0.0011 |  |  |  |  |  |
|  |  |  |  |  |  |  | (0.0146) |  |  |  |  |  |
| Financial development x ICT use |  |  |  |  |  |  |  | -0.0300 |  |  |  |  |
|  |  |  |  |  |  |  |  | (0.0424) |  |  |  |  |
| Financial development x ICT skills |  |  |  |  |  |  |  |  | -0.1626* |  |  |  |
|  |  |  |  |  |  |  |  |  | (0.0931) |  |  |  |
| Financial access x ICT access |  |  |  |  |  |  |  |  |  | 0.0130 |  |  |
|  |  |  |  |  |  |  |  |  |  | (0.0081) |  |  |
| Financial access x ICT use |  |  |  |  |  |  |  |  |  |  | 0.0139 |  |
|  |  |  |  |  |  |  |  |  |  |  | (0.0169) |  |
| Financial access x ICT skills |  |  |  |  |  |  |  |  |  |  |  | -0.3073 |
|  |  |  |  |  |  |  |  |  |  |  |  | (0.3019) |
| Constant | 0.3110*** | 0.3720*** | 0.2294*** | 0.1867** | 0.3895** | 0.5633*** | 0.1851** | 0.4283** | 0.4346* | 0.2052** | 0.3707* | 0.3643** |
|  | (0.0896) | (0.0987) | (0.0792) | (0.0789) | (0.1841) | (0.2181) | (0.0807) | (0.2175) | (0.2422) | (0.0827) | (0.1894) | (0.1855) |
| Observations | 1678 | 1,678 | 1,678 | 1636 | 608 | 1661 | 1636 | 608 | 1661 | 1,636 | 608 | 1661 |
| R-squared | 0.9408 | 0.9421 | 0.9422 | 0.9607 | 0.9079 | 0.9400 | 0.9607 | 0.9107 | 0.9388 | 0.9604 | 0.9105 | 0.9385 |
| Countries | 42 | 42 | 42 | 42 | 41 | 42 | 42 | 41 | 42 | 42 | 41 | 42 |
| Wald $X^{2}$ statistic | 10105 | 10392 | 12680 | 82034 | 31859 | 11953 | 90553 | 37551 | 19747 | 155987 | 46957 | 17483 |
| Wald P-value | 0.000 | 0.000 | 0.000 | 0.000 | 0.000 | 0.000 | 0.000 | 0.000 | 0.000 | 0.000 | 0.000 | 0.000 |
| Rho | -0.001 | -0.011 | -0.005 | 0.040 | 0.083 | 0.001 | 0.040 | 0.069 | 0.022 | 0.043 | 0.071 | 0.024 |

*Standard errors in parentheses*

**** p<0.01, ** p<0.05, * p<0.1*

***Table A.5: PCSE results on the effects of financial development, financial access, and ICTs on the severity of poverty in SSA (Dependent variable: Palma ratio)***

| Variables | (1) | (2) | (3) | (4) | (5) | (6) | (7) | (8) | (9) | (10) | (11) | (12) |
| --- | --- | --- | --- | --- | --- | --- | --- | --- | --- | --- | --- | --- |
| Palma ratio (lag) | 0.9107*** | 0.9105*** | 0.9107*** | 0.9048*** | 0.9294*** | 0.9105*** | 0.9039*** | 0.9295*** | 0.9102*** | 0.9050*** | 0.9303*** | 0.9099*** |
|  | (0.0272) | (0.0272) | (0.0273) | (0.0279) | (0.1291) | (0.0264) | (0.0280) | (0.1297) | (0.0266) | (0.0280) | (0.1291) | (0.0266) |
| Economic globalisation (KOF) | 0.0019 | 0.0015 | 0.0020 | 0.0023 | -0.0022 | 0.0015 | 0.0021 | -0.0015 | 0.0013 | 0.0022 | -0.0034 | 0.0012 |
|  | (0.0032) | (0.0033) | (0.0033) | (0.0033) | (0.0094) | (0.0038) | (0.0034) | (0.0086) | (0.0035) | (0.0034) | (0.0081) | (0.0035) |
| Social inclusion | 0.0584 | 0.0593 | 0.0585 | 0.0564 | 0.1103 | 0.0528 | 0.0540 | 0.1058 | 0.0546 | 0.0536 | 0.1250 | 0.0503 |
|  | (0.0928) | (0.0920) | (0.0935) | (0.0957) | (0.2370) | (0.1139) | (0.0975) | (0.2314) | (0.1143) | (0.0980) | (0.2409) | (0.1149) |
| Vulnerable employment | -0.0008 | -0.0006 | -0.0008 | -0.0011 | 0.0003 | -0.0005 | -0.0007 | 0.0002 | -0.0003 | -0.0007 | -0.0012 | -0.0005 |
|  | (0.0017) | (0.0016) | (0.0016) | (0.0017) | (0.0052) | (0.0017) | (0.0016) | (0.0046) | (0.0015) | (0.0020) | (0.0054) | (0.0016) |
| Foreign aid | -0.0018 | -0.0016 | -0.0019 | -0.0020 | -0.0046 | -0.0021 | -0.0015 | -0.0048 | -0.0018 | -0.0020 | -0.0041 | -0.0022 |
|  | (0.0018) | (0.0020) | (0.0018) | (0.0019) | (0.0070) | (0.0019) | (0.0020) | (0.0069) | (0.0022) | (0.0019) | (0.0065) | (0.0019) |
| GDP growth | -0.0026 | -0.0026 | -0.0026 | -0.0027 | -0.0129 | -0.0027 | -0.0029 | -0.0129 | -0.0027 | -0.0028 | -0.0130 | -0.0027 |
|  | (0.0057) | (0.0057) | (0.0057) | (0.0057) | (0.0224) | (0.0057) | (0.0057) | (0.0224) | (0.0057) | (0.0057) | (0.0225) | (0.0057) |
| Financial development |  | =0.1672 |  |  |  |  | 0.4715 | -0.1043 | 0.1898 |  |  |  |
|  |  | (0.4251) |  |  |  |  | (0.5218) | (0.6636) | (0.4356) |  |  |  |
| Financial access |  |  | -0.0136 |  |  |  |  |  |  | 0.1814 | -0.3947 | -0.9746 |
|  |  |  | (0.3714) |  |  |  |  |  |  | (0.5336) | (0.8392) | (2.2816) |
| ICT access |  |  |  | -0.0031 |  |  | 0.0040 |  |  | -0.0011 |  |  |
|  |  |  |  | (0.0088) |  |  | (0.0207) |  |  | (0.0178) |  |  |
| ICT use |  |  |  |  | -0.0246 |  |  | 0.0307 |  |  | -0.0031 |  |
|  |  |  |  |  | (0.0447) |  |  | (0.0752) |  |  | (0.0651) |  |
| ICT skills |  |  |  |  |  | -0.0276 |  |  | -0.0264 |  |  | -0.0434 |
|  |  |  |  |  |  | (0.0308) |  |  | (0.0311) |  |  | (0.0325) |
| Financial development x ICT access |  |  |  |  |  |  | -0.0349 |  |  |  |  |  |
|  |  |  |  |  |  |  | (0.0551) |  |  |  |  |  |
| Financial development x ICT use |  |  |  |  |  |  |  | -0.0228 |  |  |  |  |
|  |  |  |  |  |  |  |  | (0.1778) |  |  |  |  |
| Financial development x ICT skills |  |  |  |  |  |  |  |  | -0.0068 |  |  |  |
|  |  |  |  |  |  |  |  |  | (0.0804) |  |  |  |
| Financial access x ICT access |  |  |  |  |  |  |  |  |  | -0.0109 |  |  |
|  |  |  |  |  |  |  |  |  |  | (0.0420) |  |  |
| Financial access x ICT use |  |  |  |  |  |  |  |  |  |  | -0.0900 |  |
|  |  |  |  |  |  |  |  |  |  |  | (0.1395) |  |
| Financial access x ICT skills |  |  |  |  |  |  |  |  |  |  |  | -0.1542 |
|  |  |  |  |  |  |  |  |  |  |  |  | (0.3527) |
| Constant | 0.4726 | 0.4483 | 0.4715 | 0.5298 | 0.3467 | 0.6610 | 0.4653 | 0.3566 | 0.6194 | 0.5083 | 0.4812 | 0.7924 |
|  | (0.4145) | (0.3971) | (0.4202) | (0.4134) | (1.5098) | (0.5746) | (0.3927) | (1.4005) | (0.5691) | (0.4135) | (1.4949) | (0.5767) |
| Observations | 1680 | 1,680 | 1680 | 1638 | 610 | 1663 | 1638 | 610 | 1663 | 1,638 | 610 | 1663 |
| R-squared | 0.8307 | 0.8304 | 0.8307 | 0.8205 | 0.7183 | 0.8310 | 0.8195 | 0.7186 | 0.8308 | 0.8207 | 0.7204 | 0.8299 |
| Countries | 42 | 42 | 42 | 42 | 41 | 42 | 42 | 41 | 42 | 42 | 41 | 42 |
| Wald $X^{2}$ statistic | 1314 | 1317 | 1364 | 1280 | 588.5 | 3062 | 1304 | 589.7 | 3143 | 1377 | 673.4 | 4104 |
| Wald P-value | 0.000 | 0.000 | 0.000 | 0.000 | 0.000 | 0.000 | 0.000 | 0.000 | 0.000 | 0.000 | 0.000 | 0.000 |
| Rho | 0.171 | 0.172 | 0.172 | 0.175 | 0.121 | 0.170 | 0.177 | 0.120 | 0.170 | 0.174 | 0.118 | 0.172 |

*Standard errors in parentheses*

**** p<0.01, ** p<0.05, * p<0.1*

***Table A.6: PCSE results on the effects of financial development, financial access, and ICTs on the intensity of poverty in SSA (Dependent variable: Poverty gap US$1.90)***

| Variables | (1) | (2) | (3) | (4) | (5) | (6) | (7) | (8) | (9) | (10) | (11) | (12) |
| --- | --- | --- | --- | --- | --- | --- | --- | --- | --- | --- | --- | --- |
| Poverty gap US$1.90 (lag) | 0.9671*** | 0.9652*** | 0.9484*** | 0.9738*** | 0.9607*** | 0.9616*** | 0.9738*** | 0.9613*** | 0.9327*** | 0.9718*** | 0.9528*** | 0.9318*** |
|  | (0.0122) | (0.0123) | (0.0118) | (0.0073) | (0.0277) | (0.0191) | (0.0073) | (0.0290) | (0.0207) | (0.0079) | (0.0319) | (0.0215) |
| Economic globalisation (KOF) | -0.0033*** | -0.0029*** | -0.0017*** | -0.0008* | -0.0023 | -0.0038*** | -0.0008* | -0.0018 | -0.0020** | -0.0008 | -0.0013 | -0.0019** |
|  | (0.0008) | (0.0007) | (0.0006) | (0.0005) | (0.0015) | (0.0010) | (0.0005) | (0.0016) | (0.0009) | (0.0005) | (0.0016) | (0.0009) |
| Social inclusion | 0.0041 | 0.0029 | 0.0079 | -0.0089 | -0.0163 | -0.0008 | -0.0089 | -0.0205 | 0.0050 | -0.0067 | -0.0149 | 0.0059 |
|  | (0.0117) | (0.0116) | (0.0115) | (0.0109) | (0.0291) | (0.0135) | (0.0109) | (0.0280) | (0.0137) | (0.0110) | (0.0293) | (0.0142) |
| Vulnerable employment | -0.0004 | -0.0006** | -0.0003 | -0.0001 | -0.0002 | -0.0001 | -0.0001 | -0.0004 | 0.0004 | -0.0004 | -0.0004 | 0.0004 |
|  | (0.0002) | (0.0003) | (0.0002) | (0.0002) | (0.0005) | (0.0004) | (0.0002) | (0.0005) | (0.0004) | (0.0002) | (0.0005) | (0.0005) |
| Foreign aid | 0.0003 | 0.0000 | 0.0003 | 0.0002 | 0.0004 | 0.0002 | 0.0002 | 0.0001 | 0.0002 | 0.0002 | 0.0005 | 0.0002 |
|  | (0.0007) | (0.0007) | (0.0007) | (0.0005) | (0.0021) | (0.0009) | (0.0005) | (0.0022) | (0.0009) | (0.0005) | (0.0021) | (0.0009) |
| GDP growth | -0.0008 | -0.0008 | -0.0010 | -0.0016* | -0.0025 | -0.0008 | -0.0016* | -0.0025 | -0.0012 | -0.0015* | -0.0028 | -0.0013 |
|  | (0.0013) | (0.0013) | (0.0013) | (0.0009) | (0.0041) | (0.0017) | (0.0009) | (0.0041) | (0.0017) | (0.0009) | (0.0040) | (0.0016) |
| Financial development |  | -0.1976** |  |  |  |  | 0.0001 | -0.1122 | 0.0577 |  |  |  |
|  |  | (0.0831) |  |  |  |  | (0.0852) | (0.2231) | (0.1807) |  |  |  |
| Financial access |  |  | -0.3707** |  |  |  |  |  |  | -0.1256* | -0.2287 | 0.5331 |
|  |  |  | (0.1731) |  |  |  |  |  |  | (0.0656) | (0.1873) | (0.9487) |
| ICT access |  |  |  | -0.0040*** |  |  | -0.0040* |  |  | -0.0061*** |  |  |
|  |  |  |  | (0.0014) |  |  | (0.0021) |  |  | (0.0020) |  |  |
| ICT use |  |  |  |  | -0.0035 |  |  | -0.0007 |  |  | -0.0030 |  |
|  |  |  |  |  | (0.0064) |  |  | (0.0036) |  |  | (0.0050) |  |
| ICT skills |  |  |  |  |  | -0.0179 |  |  | -0.0226 |  |  | -0.0154 |
|  |  |  |  |  |  | (0.0170) |  |  | (0.0173) |  |  | (0.0118) |
| Financial development x ICT access |  |  |  |  |  |  | -0.0000 |  |  |  |  |  |
|  |  |  |  |  |  |  | (0.0077) |  |  |  |  |  |
| Financial development x ICT use |  |  |  |  |  |  |  | -0.0097 |  |  |  |  |
|  |  |  |  |  |  |  |  | (0.0249) |  |  |  |  |
| Financial development x ICT skills |  |  |  |  |  |  |  |  | -0.0758 |  |  |  |
|  |  |  |  |  |  |  |  |  | (0.0473) |  |  |  |
| Financial access x ICT access |  |  |  |  |  |  |  |  |  | -0.0084** |  |  |
|  |  |  |  |  |  |  |  |  |  | (0.0042) |  |  |
| Financial access x ICT use |  |  |  |  |  |  |  |  |  |  | -0.0074 |  |
|  |  |  |  |  |  |  |  |  |  |  | (0.0093) |  |
| Financial access x ICT skills |  |  |  |  |  |  |  |  |  |  |  | -0.1561 |
|  |  |  |  |  |  |  |  |  |  |  |  | (0.1607) |
| Constant | 0.2273*** | 0.2631*** | 0.2293*** | 0.1466*** | 0.2646** | 0.3705*** | 0.1466*** | 0.2846** | 0.3893** | 0.1673*** | 0.2756** | 0.3424*** |
|  | (0.0478) | (0.0545) | (0.0495) | (0.0414) | (0.1085) | (0.1420) | (0.0420) | (0.1228) | (0.1594) | (0.0429) | (0.1101) | (0.1128) |
| Observations | 1,678 | 1678 | 1,678 | 1,636 | 608 | 1661 | 1,636 | 608 | 1,661 | 1,636 | 608 | 1661 |
| R-squared | 0.9465 | 0.9472 | 0.9472 | 0.9656 | 0.9207 | 0.9457 | 0.9656 | 0.9225 | 0.9437 | 0.9651 | 0.9201 | 0.9432 |
| Countries | 42 | 42 | 42 | 42 | 41 | 42 | 42 | 41 | 42 | 42 | 41 | 42 |
| Wald $X^{2}$ statistic | 13148 | 13446 | 16330 | 100760 | 38644 | 15023 | 116220 | 45248 | 24235 | 194207 | 55736 | 20980 |
| Wald P-value | 0.000 | 0.000 | 0.000 | 0.000 | 0.000 | 0.000 | 0.000 | 0.000 | 0.000 | 0.000 | 0.000 | 0.000 |
| Rho | -0.010 | -0.017 | -0.011 | 0.014 | 0.049 | -0.006 | 0.014 | 0.037 | 0.020 | 0.021 | 0.054 | 0.025 |

*Standard errors in parentheses*

**** p<0.01, ** p<0.05, * p<0.1*

***Table A.7: PCSE results on the effects of financial development, financial access, and ICTs on the intensity of poverty in SSA (Dependent variable: Poverty gap US$3.20)***

| Variables | (1) | (2) | (3) | (4) | (5) | (6) | (7) | (8) | (9) | (10) | (11) | (12) |
| --- | --- | --- | --- | --- | --- | --- | --- | --- | --- | --- | --- | --- |
| Poverty US$3.20 (lag) | 0.9589*** | 0.9571*** | 0.9195*** | 0.9825*** | 0.9783*** | 0.9564*** | 0.9774*** | 0.9847*** | 0.8986*** | 0.9709*** | 0.9609*** | 0.9001*** |
|  | (0.0181) | (0.0183) | (0.0222) | (0.0186) | (0.0535) | (0.0248) | (0.0193) | (0.0520) | (0.0326) | (0.0200) | (0.0593) | (0.0329) |
| Economic globalisation (KOF) | -0.0020*** | -0.0017*** | -0.0003 | -0.0004 | -0.0016 | -0.0022** | -0.0003 | -0.0025 | -0.0005 | 0.0001 | 0.0001 | -0.0004 |
|  | (0.0007) | (0.0006) | (0.0007) | (0.0005) | (0.0020) | (0.0010) | (0.0005) | (0.0019) | (0.0010) | (0.0005) | (0.0021) | (0.0010) |
| Social inclusion | 0.0051 | 0.0042 | 0.0128 | -0.0043 | -0.0002 | 0.0020 | 0.0027 | -0.0020 | 0.0142 | 0.0000 | 0.0018 | 0.0130 |
|  | (0.0078) | (0.0076) | (0.0088) | (0.0079) | (0.0227) | (0.0094) | (0.0095) | (0.0227) | (0.0113) | (0.0086) | (0.0225) | (0.0108) |
| Vulnerable employment | -0.0003 | -0.0005 | -0.0002 | -0.0001 | -0.0005 | -0.0002 | -0.0002 | -0.0011 | 0.0005 | -0.0002 | -0.0009 | 0.0003 |
|  | (0.0004) | (0.0004) | (0.0004) | (0.0003) | (0.0014) | (0.0007) | (0.0004) | (0.0019) | (0.0008) | (0.0004) | (0.0013) | (0.0008) |
| Foreign aid | 0.0003 | 0.0001 | 0.0003 | 0.0001 | -0.0006 | 0.0002 | -0.0000 | -0.0007 | 0.0004 | 0.0002 | -0.0003 | 0.0002 |
|  | (0.0005) | (0.0005) | (0.0005) | (0.0003) | (0.0016) | (0.0006) | (0.0004) | (0.0016) | (0.0006) | (0.0003) | (0.0016) | (0.0007) |
| GDP growth | -0.0003 | -0.0003 | -0.0005 | -0.0011 | -0.0013 | -0.0003 | -0.0009 | -0.0010 | -0.0007 | -0.0011 | -0.0019 | -0.0007 |
|  | (0.0011) | (0.0011) | (0.0011) | (0.0007) | (0.0034) | (0.0014) | (0.0007) | (0.0034) | (0.0013) | (0.0007) | (0.0033) | (0.0013) |
| Financial development |  | -0.1478** |  |  |  |  | -0.2718 | -0.3580 | 0.1904 |  |  |  |
|  |  | (0.0719) |  |  |  |  | (0.1708) | (0.3536) | (0.1329) |  |  |  |
| Financial access |  |  | -0.4852*** |  |  |  |  |  |  | -0.2876** | -0.4113 | 0.4013 |
|  |  |  | (0.1629) |  |  |  |  |  |  | (0.1334) | (0.3069) | (0.8997) |
| ICT access |  |  |  | 0.0014 |  |  | -0.0129 |  |  | 0.0018 |  |  |
|  |  |  |  | (0.0037) |  |  | (0.0092) |  |  | (0.0068) |  |  |
| ICT use |  |  |  |  | 0.0178 |  |  | -0.0167 |  |  | 0.0200 |  |
|  |  |  |  |  | (0.0211) |  |  | (0.0322) |  |  | (0.0266) |  |
| ICT skills |  |  |  |  |  | -0.0081 |  |  | -0.0148 |  |  | -0.0100 |
|  |  |  |  |  |  | (0.0192) |  |  | (0.0198) |  |  | (0.0128) |
| Financial development x ICT access |  |  |  |  |  |  | 0.0510* |  |  |  |  |  |
|  |  |  |  |  |  |  | (0.0306) |  |  |  |  |  |
| Financial development x ICT use |  |  |  |  |  |  |  | 0.1440 |  |  |  |  |
|  |  |  |  |  |  |  |  | (0.1183) |  |  |  |  |
| Financial development x ICT skills |  |  |  |  |  |  |  |  | -0.1029** |  |  |  |
|  |  |  |  |  |  |  |  |  | (0.0417) |  |  |  |
| Financial access x ICT access |  |  |  |  |  |  |  |  |  | 0.0064 |  |  |
|  |  |  |  |  |  |  |  |  |  | (0.0104) |  |  |
| Financial access x ICT use |  |  |  |  |  |  |  |  |  |  | 0.0071 |  |
|  |  |  |  |  |  |  |  |  |  |  | (0.0312) |  |
| Financial access x ICT skills |  |  |  |  |  |  |  |  |  |  |  | -0.1538 |
|  |  |  |  |  |  |  |  |  |  |  |  | (0.1529) |
| Constant | 0.2201*** | 0.2506*** | 0.2950*** | 0.0910 | 0.1682 | 0.2938* | 0.1346 | 0.2866 | 0.3979** | 0.1222 | 0.2138 | 0.3930*** |
|  | (0.0761) | (0.0845) | (0.0901) | (0.0803) | (0.1892) | (0.1620) | (0.0845) | (0.2416) | (0.1858) | (0.0863) | (0.2020) | (0.1442) |
| Observations | 1680 | 1,680 | 1680 | 1638 | 610 | 1663 | 1,638 | 610 | 1663 | 1,638 | 610 | 1,663 |
| R-squared | 0.9362 | 0.9379 | 0.9326 | 0.9589 | 0.9013 | 0.9371 | 0.9574 | 0.8990 | 0.9283 | 0.9572 | 0.9078 | 0.9284 |
| Countries | 42 | 42 | 42 | 42 | 41 | 42 | 42 | 41 | 42 | 42 | 41 | 42 |
| Wald $X^{2}$ statistic | 4396 | 4607 | 4230 | 17287 | 1241 | 2653 | 37524 | 3532 | 2445 | 38115 | 3082 | 2397 |
| Wald P-value | 0.000 | 0.000 | 0.000 | 0.000 | 0.000 | 0.000 | 0.000 | 0.000 | 0.000 | 0.000 | 0.000 | 0.000 |
| Rho | -0.022 | -0.035 | 0.018 | -0.009 | 0.029 | -0.032 | 0.015 | 0.058 | 0.051 | 0.014 | 0.003 | 0.049 |

*Standard errors in parentheses*

**** p<0.01, ** p<0.05, * p<0.1*
